# Acylation of the Incretin Peptide Exendin-4 Directly Impacts Glucagon-Like Peptide-1 Receptor Signaling and Trafficking[Fn fn4]

**DOI:** 10.1124/molpharm.121.000270

**Published:** 2021-10

**Authors:** Maria Lucey, Tanyel Ashik, Amaara Marzook, Yifan Wang, Joëlle Goulding, Atsuro Oishi, Johannes Broichhagen, David J. Hodson, James Minnion, Yuval Elani, Ralf Jockers, Stephen J. Briddon, Stephen R. Bloom, Alejandra Tomas, Ben Jones

**Affiliations:** Section of Endocrinology and Investigative Medicine (M.L., T.A., A.M., J.M., S.R.B., B.J.) and Section of Cell Biology and Functional Genomics (Y.W., A.T.), Department of Metabolism, Digestion and Reproduction, and Department of Chemical Engineering (Y.E.), Imperial College London, London, United Kingdom; Division of Physiology, Pharmacology and Neuroscience, School of Life Sciences, University of Nottingham, Nottingham, United Kingdom (J.G., S.J.B.); Centre of Membrane Proteins and Receptors (COMPARE), University of Birmingham and University of Nottingham, Midlands, United Kingdom (J.G., D.J.H., S.J.B.); Université de Paris, Institut Cochin, INSERM, CNRS, Paris, France (A.O., R.J.); Department of Anatomy, Kyorin University Faculty of Medicine, Tokyo, Japan (A.O.); Leibniz-Forschungsinstitut für Molekulare Pharmakologie, Berlin, Germany (J.B.); Institute of Metabolism and Systems Research (IMSR), University of Birmingham, Birmingham, United Kingdom (D.J.H.); and Centre for Endocrinology, Diabetes and Metabolism, Birmingham Health Partners, Birmingham, United Kingdom (D.J.H.)

## Abstract

The glucagon-like peptide-1 receptor (GLP-1R) is a class B G protein–coupled receptor and mainstay therapeutic target for the treatment of type 2 diabetes and obesity. Recent reports have highlighted how biased agonism at the GLP-1R affects sustained glucose-stimulated insulin secretion through avoidance of desensitization and downregulation. A number of GLP-1R agonists (GLP-1RAs) feature a fatty acid moiety to prolong their pharmacokinetics via increased albumin binding, but the potential for these chemical changes to influence GLP-1R function has rarely been investigated beyond potency assessments for cAMP. Here, we directly compare the prototypical GLP-1RA exendin-4 with its C-terminally acylated analog, exendin-4-C16. We examine relative propensities of each ligand to recruit and activate G proteins and *β*-arrestins, endocytic and postendocytic trafficking profiles, and interactions with model and cellular membranes in HEK293 and HEK293T cells. Both ligands had similar cAMP potency, but exendin-4-C16 showed ∼2.5-fold bias toward G protein recruitment and a ∼60% reduction in *β*-arrestin-2 recruitment efficacy compared with exendin-4, as well as reduced GLP-1R endocytosis and preferential targeting toward recycling pathways. These effects were associated with reduced movement of the GLP-1R extracellular domain measured using a conformational biosensor approach and a ∼70% increase in insulin secretion in INS-1 832/3 cells. Interactions with plasma membrane lipids were enhanced by the acyl chain. Exendin-4-C16 showed extensive albumin binding and was highly effective for lowering of blood glucose in mice over at least 72 hours. Our study highlights the importance of a broad approach to the evaluation of GLP-1RA pharmacology.

**SIGNIFICANCE STATEMENT:**

Acylation is a common strategy to enhance the pharmacokinetics of peptide-based drugs. This work shows how acylation can also affect various other pharmacological parameters, including biased agonism, receptor trafficking, and interactions with the plasma membrane, which may be therapeutically important.

## Introduction

Type 2 diabetes (T2D) affects over 400 million people worldwide and leads to chronic illnesses including blindness, nerve damage, kidney failure, and cardiac disease (Guariguata et al., 2014). Pathologic increases in blood glucose, the central metabolic defect in T2D, occur chiefly because of failure of pancreatic *β* cells to release enough of the glucoregulatory hormone insulin and overweight-related tissue resistance to insulin action. Therefore, pharmacological targeting of the glucagon-like peptide-1 receptor (GLP-1R), a class B G protein–coupled receptor (GPCR) that increases insulin release in a glucose-dependent manner and also suppresses appetite, leading to weight loss, is an effective strategy against T2D (Graaf et al., 2016). As GLP-1(7-36)NH_2_, the endogenous ligand for GLP-1R, is enzymatically inactivated in the circulation within minutes and eliminated by glomerular filtration, a number of strategies have been used to develop pharmaceutically viable peptide GLP-1R agonists (GLP-1RAs) that remain active in the circulation for hours or days (Andersen et al., 2018). As typified by exendin-4 (in clinical use as exenatide), the peptide amino acid sequence can be altered to prevent proteolytic degradation, extending circulatory half-life from minutes to hours while retaining full activity at the receptor (Kolterman et al., 2003). An alternative or complementary approach, the best known exemplars of which are liraglutide and semaglutide (Lau et al., 2015), involves attachment of a fatty acid side chain to the peptide to allow it to bind reversibly to albumin. Specifically, liraglutide includes a palmitic (C16) acid chain, which was switched to a C18 fatty diacid chain in semaglutide to improve affinity for human albumin (Knudsen and Lau, 2019). As albumin exceeds the size limit for glomerular filtration, this major elimination route is lost (Malm-Erjefält et al., 2010). Half-life protraction of semaglutide via this strategy allows once-weekly administration in humans (Knudsen and Lau, 2019).

Although GLP-1RA pharmacology has been traditionally assessed by measuring cAMP responses, additional factors are now recognized as important determinants of insulin release. For example, changes to the amino acid sequence of GLP-1R peptide agonists in their receptor core-facing N-terminal region can lead to bias between G protein signaling, *β*-arrestin recruitment, and receptor endocytosis (Zhang et al., 2015; Jones et al., 2018). We recently reported how a dual strategy based on both pharmacokinetically preferential acylation and G protein–directed biased agonism could optimize GLP-1R therapeutic efficacy (Lucey et al., 2020). Although not explored in detail in the latter project, we observed that the addition of a C-terminal acyl chain further influenced the ability of each ligand to recruit G proteins and *β*-arrestins to the GLP-1R, highlighting this feature as a modifier of GLP-1R efficacy in its own right.

Moreover, it is conceivable that acylation could constructively influence the interaction between agonist and plasma membrane by conferring distinct physicochemical properties, as suggested for the acylated analogs of GLP-2 (Trier et al., 2014). This has the potential to modulate downstream signaling by directing the ligand toward GLP-1Rs situated in submicroscopic membrane domains variably enriched with signaling effectors on the cytoplasmic side (Villar et al., 2016). Indeed, differences in segregation of activated GLP-1Rs into these “membrane rafts” are an important feature of biased agonist action (Buenaventura et al., 2019).

To investigate these possibilities, in this study we performed a detailed comparison of the prototypical GLP-1R therapeutic agonist exendin-4 with an equivalent peptide bearing a C16 fatty diacid at its C terminus, focusing on the relative propensities of each ligand to engage with G protein and *β*-arrestin recruitment and activation, endocytosis, and postendocytic sorting in HEK293 and HEK293T cells. We also evaluated interactions made by each ligand with giant unilamellar vesicles (GUVs) and the relative impact of modulating cellular cholesterol levels on GLP-1R signaling with each ligand. Our study highlights how installation of an acyl chain at the C terminus influences multiple pharmacological properties of exendin-4, enhancing its ability to stimulate insulin release from pancreatic *β* cells.

## Materials and Methods

### Peptides and Reagents

All peptides were obtained from Wuxi Apptec (Wuhan, China) and were at least 90% pure. Laboratory reagents were obtained from Sigma Aldrich (Dorset, UK) unless otherwise specified.

### Cell Culture

HEK293T cells were maintained in Dulbecco’s modified Eagle’s medium (DMEM) supplemented with 10% FBS and 1% penicillin/streptomycin. HEK293-SNAP-GLP-1R cells generated by stable transfection of pSNAP-GLP-1R (Cisbio, Paris, France) into HEK293 cells (Buenaventura et al., 2019) were used for some experiments and maintained in DMEM supplemented with 10% FBS, 1% penicillin/streptomycin, and 1 mg/ml G418. T-REx-SNAP-GLP-1R cells (Fang et al., 2020a), generated from parental Flp-In T-REx 293 cells (Thermo Fisher Scientific, MA) by Flp-mediated genomic integration of an insert encoding human GLP-1R with an N-terminal SNAP_f_-tag and C-terminal SmBiT tag (custom-synthesized by Genewiz, Steißlingen, Germany), with pcDNA5/FRT as the backbone, were maintained in DMEM supplemented with 10% FBS and 1% penicillin/streptomycin. SNAP-GLP-1R expression was induced using 0.1 µg/ml tetracycline for 24 hours prior to experiments. PathHunter GLP-1R *β*-arrestin-1 and -2 cells (DiscoverX) were maintained in Ham’s F12 medium with 10% FBS and 1% penicillin/streptomycin. INS-1 832/3 cells (a gift from Prof. Christopher Newgard, Duke University) (Hohmeier et al., 2000) were maintained in RPMI at 11 mM glucose, supplemented with 10% FBS, 10 mM HEPES, 1 mM pyruvate, 1 mM pyruvate, 50 μM *β*-mercaptoethanol, and 1% penicillin/streptomycin.

### Equilibrium GLP-1R Binding Assays

HEK293-SNAP-GLP-1R cells were labeled using 40 nM SNAP-Lumi4-Tb (Cisbio) in complete medium for 60 minutes at room temperature. After washing, cells were resuspended in Hanks’ balanced salt solution (HBSS) containing 0.1% BSA and metabolic inhibitors (10 mM NaN_3_ and 20 mM 2-deoxyglucose) to prevent endocytosis (Widmann et al., 1995). After 20 minutes at room temperature, cells in 96-well white plates were equilibrated to 4°C before addition of agonists. To measure binding parameters for exendin-4 and exendin-4-C16, these nonlabeled ligands were added alongside 10 nM exendin(9-39)-FITC and incubated for 24 hours in the dark to allow binding equilibrium to be achieved. TR-FRET signal from each well was then recorded using a Flexstation 3 with the following settings: *λ*_ex_ 340 nm, *λ*_em_ 520 and 620 nm, autocutoff, delay 50 µs, integration time 300 µs. A saturation binding isotherm for exendin(9-39)-FITC was performed during each experiment, and the K_i_ values for competing agonists were determined using the “Fit K_i_” preset of Prism 8.0. To measure saturation binding for FITC- or TMR-labeled exendin-4 and exendin-4-C16 directly, these ligands were added to cells and incubated as above, followed by measurement of TR-FRET signal using the following settings: FITC ligands: *λ*_ex_ 340 nm, *λ*_em_ 520 and 620 nm, autocutoff, delay 50 µs, integration time 300 µs; TMR ligands: *λ*_ex_ 340 nm, *λ*_em_ 550 and 610 nm, autocutoff, delay 50 µs, integration time 300 µs.

### Measurement of cAMP Production

HEK293-SNAP-GLP-1R cells were resuspended in serum-free medium and treated at 37°C with indicated concentration of agonist for 30 minutes. cAMP was then assayed by HTRF (Cisbio cAMP Dynamic 2) using a Spectramax i3x plate reader (Molecular Devices). Where indicated, cells were preincubated with methyl-*β*-cyclodextrin in advance of the assay before washing.

### NanoBiT Assay

The assay was performed as previously described (Lucey et al., 2020). HEK293T cells in 12-well plates were transfected with 0.5 µg GLP-1R-SmBiT plus 0.5 µg LgBiT-mini-G_s_ (Wan et al., 2018) (a gift from Prof. Nevin Lambert, Medical College of Georgia), or with 0.05 µg GLP-1R-SmBiT and 0.05 µg LgBit-*β*-arrestin-2 (Promega, Hampshire, UK) plus 0.9 µg pcDNA3.1 for 24 hours. Cells were detached with EDTA and resuspended in HBSS, and furimazine (Promega) was added at a 1:50 dilution from the manufacturer’s preprepared stock. After dispensing into 96-well white plates, a baseline read of luminescent signal was serially recorded over 5 minutes using a Flexstation 3 instrument (Molecular Devices) at 37°C before addition of the indicated concentration of ligand, after which the signal was repeatedly recorded for 30 minutes. Results were expressed relative to individual well baseline, and AUC was calculated. Each assay was performed with both pathways measured in parallel using the same ligand stocks. Bias between mini-G_s_ and *β*-arrestin-2 recruitment was determined similarly to earlier work (Pickford et al., 2020), by subtracting the logτ /K_A_ values (Kenakin et al., 2012) for each ligand in each pathway for each assay to determine Δ logτ /K_A_ (referred to here as Δ log R); this indicates the relative pathway preference for each ligand, which can then be compared statistically.

### ***β***-Arrestin-1 and -2 Recruitment by Enzyme Complementation

PathHunter GLP-1R *β*-arrestin-1 or -2 cells were treated in serum-free medium for 30 minutes with a range of agonist concentrations before addition of PathHunter chemiluminescent detection reagents. Bias was assessed as in the section *NanoBiT Assay*.

### ***β***-Arrestin Conformational Change Assay

The assay was adapted from a previous description (Oishi et al., 2019). HEK293T cells were seeded in six-well plates and transfected with 0.5 µg NLuc-*β*-arrestin-2-CyOFP under a TK promoter, 0.5 µg SNAP-GLP-1R under a CMV promoter, plus 1 µg pcDNA3.1, for 24 hours before the assay. Alternatively, stable HEK293-SNAP-GLP-1R cells in six-well plates were transfected with 0.5 µg NLuc-*β*-arrestin-2-CyOFP plus 1.5 µg pcDNA3.1. Cells were detached with EDTA and resuspended in HBSS with furimazine (1:50 dilution). After dispensing into 96-well white plates, a baseline read of luminescent signals at both 460 nm and 575 nm was serially recorded over 5 minutes using a Flexstation 3 instrument at 37°C. Ligands in HBSS were then added, after which signal was repeatedly recorded for 30 minutes. Results were first normalized to individual well baseline and then to the average vehicle signal within each assay (see Supplemental Fig. 5A). Statistical comparisons were performed on AUC calculated from each ligand-induced kinetic trace.

### G Protein Activation Assay by Nb37 BRET

A construct encoding SNAP-GLP-1R with a C-terminal nanoluciferase was generated in-house by PCR cloning of the nanoluciferase sequence from pcDNA3.1-ccdB-Nanoluc (a gift from Mikko Taipale; Addgene plasmid # 87067) onto the C terminus end of the SNAP-GLP-1R vector (Cisbio), followed by site-directed mutagenesis of the GLP-1R stop codon. HEK293T cells in six-well plates were transfected with 0.2 µg SNAP-GLP-1R-NLuc, 0.2 µg Nb37-GFP (a gift from Dr Roshanak Irranejad, UCSF), and 1.6 µg pcDNA3.1 for 24 hours before the assay. Cells were detached with EDTA and resuspended in HBSS, and furimazine was added at a 1:50 dilution from the manufacturer’s preprepared stock. After dispensing into 96-well white plates, a baseline read of luminescent signals at both 460 nm and 525 nm was serially recorded over 5 minutes using a Flexstation 3 instrument at 37°C. Ligands in HBSS were then added, after which signal was repeatedly recorded for 30 minutes. Results were first normalized to individual well baseline and then to the average vehicle signal from each assay (see Supplemental Fig. 5B). Statistical comparisons were performed on AUC calculated from each ligand-induced kinetic trace.

### Preparation and Imaging of Fixed Cell Samples

Cells were seeded onto coverslips coated with 0.1% poly(d-lysine) and allowed to adhere overnight. Where relevant, transfection was performed as for functional assays, and surface labeling of SNAP-tagged GLP-1R was performed using 0.5 µM of the indicated SNAP-Surface probe for 30 minutes at 37°C before washing with HBSS. Ligands were applied in Ham’s F12 media containing 0.1% BSA at 37°C. For fixation, 4% paraformaldehyde was applied directly to the medium for 15 minutes before washing with PBS. Slides were mounted in Prolong Diamond antifade with DAPI (Thermo Fisher Scientific) and allowed to set overnight. Imaging was performed using a Nikon Ti2E custom microscope platform with automated stage (ASI) and LED light source controlled by µManager. High-resolution z-stacks were acquired via a 100× 1.45 NA oil immersion objective and used for image deconvolution using DeconvolutionLab2 (Sage et al., 2017) with the Richardson-Lucy algorithm.

### Measurement of GLP-1R Internalization by Time-Lapse High-Content Microscopy

HEK293-SNAP-GLP-1R cells were seeded overnight in black, clear-bottom 96-well imaging plates coated with 0.1% poly(d-lysine). Cells were labeled with SNAP-Surface-649 (0.5 µM, from New England Biolabs, Hitchin, UK) for 30 minutes at 37°C. After washing, imaging medium was added to the wells (phenol red-free DMEM with 10 mM HEPES and 0.1% BSA) at 37°C. Baseline epifluorescence and transmitted phase contrast images were acquired using a 20× magnification 0.75 NA objective from four positions per well using the imaging system in the section *G Protein Activation Assay by Nb37 BRET*. Further baseline epifluorescence images were acquired using a 40× magnification 0.95 NA objective, after which ligands were added directly to each well. Images in the same four positions were acquired every 3 minutes for 30 minutes. To quantify the translocation of surface-labeled SNAP-GLP-1R from plasma membrane to endosomes, a “spot-counting” imaging-processing pipeline implemented in Fiji was developed as follows: 1) flatfield correction was applied to the epifluorescence images using BaSiC (Peng et al., 2017); 2) Laplacian filtering (smoothening scale: 2.0) was applied using FeatureJ; 3) starting with an image in which multiple endosomal puncta were visible, images were thresholded using the autoselected threshold from the “Triangle” algorithm, and the same threshold was used for all images; 4) spots were counted using the particle-counting algorithm in Fiji with a size limit of 0.5–5 µm and roundness of 0.5–1; 5) differences in cell density within each image were accounted for by estimating confluence from the phase contrast image (cropped to the region covered by the 40× objective) using PHANTAST (Jaccard et al., 2014) and dividing the spot count by this value; 6) after normalization, the number of spots present at baseline was subtracted to identify the ligand-induced changes. Rate constants were determined from one-phase exponential curve fitting in Prism 8.0.

### Measurement of GLP-1R Internalization by DERET

The assay was performed as previously described (Lucey et al., 2020). HEK-SNAP-GLP-1R cells were labeled using 40 nM SNAP-Lumi4-Tb in complete medium for 60 minutes at room temperature. After washing, cells were resuspended in HBSS containing 24 µM fluorescein and dispensed into 96-well white plates. A baseline read was serially recorded over 5 minutes using a Flexstation 3 instrument at 37°C in TR-FRET mode with the following settings: *λ*_ex_ 340 nm, *λ*_em_ 520 and 620 nm, autocutoff, delay 400 µs, integration time 1500 µs. Ligands were then added, after which the signal was repeatedly recorded for 30 minutes. Fluorescence signals were expressed ratiometrically after first subtracting signal from wells containing 24 µM fluorescein without cells. Internalization was quantified as AUC relative to individual well baseline.

### Measurement of GLP-1R Trafficking to Acidic Endosomes Using LysoTracker

HEK-SNAP-GLP-1R cells were labeled with SNAP-Lumi4-Tb (40 nM, 60 minutes at 37°C, in complete medium), with LysoTracker Red DND99 (100 nM) added for the last 15 minutes of the incubation period. After washing, labeled cells were resuspended in HBSS. TR-FRET signals at baseline and serially after agonist addition were recorded at 37°C using a Flexstation 3 plate reader using the following settings: *λ*_ex_ = 335 nm, *λ*_em_ = 550 and 610 nm, delay 50 μs, integration time 300 μs. Receptor translocation to acidic endosomes was quantified as the ratio of fluorescent signal at 610 nm to that at 550 nm.

### Measurement of Receptor Recycling by TR-FRET

The assay was performed as previously described (Fang et al., 2020b). Adherent HEK293-SNAP-GLP-1R cells in 96-well white plates coated with 0.1% poly(d-lysine) were labeled with 40 nM BG-S-S-Lumi4-Tb in complete medium for 60 minutes at room temperature. After washing, cells were treated for 30 minutes with each agonist at 100 nM or vehicle (serum-free medium) to induce GLP-1R internalization. Cells were then washed once with HBSS before application of alkaline TNE buffer (pH 8.6) containing (or not) 100 mM Mesna for 5 minutes at 4°C to cleave residual surface GLP-1R. In total, 10 nM Luxendin645 (Ast et al., 2020) in HBSS containing 0.1% BSA was then added, and TR-FRET signal was serially monitored using a Spectramax i3x multimode plate reader with HTRF module. Ratiometric HTRF responses were normalized to baseline from the first three reads.

### Measurement of GLP-1R Compartmentalization by BRET

HEK293T cells in six-well plates were transfected with 0.2 µg SNAP-GLP-1R-NLuc, 0.2 µg of KRAS-Venus, Rab5-Venus, Rab7-Venus, or Rab11-Venus (kindly provided by Prof. Nevin Lambert, Medical College of Georgia, and Prof. Kevin Pfleger, University of Western Australia), plus 1.6 µg pcDNA3.1 24 hours before the assay. Cells were detached with EDTA and resuspended in HBSS, and furimazine was added at a 1:50 dilution from the manufacturer’s preprepared stock. After dispensing into 96-well white plates, a baseline read of luminescent signals at both 460 nm and 535 nm was serially recorded over 5 minutes using a Flexstation 3 instrument at 37°C. Ligands in HBSS were then added, after which signal was repeatedly recorded for 30 minutes. Results were first normalized to individual well baseline and then to the average vehicle signal from each experiment (see Supplemental Fig. 5, C–F). Statistical comparisons were performed on AUC calculated from each ligand-induced kinetic trace.

### Mini-G_s_ Subcellular Translocation Assay

HEK293-SNAP-GLP-1R cells were seeded in six-well plates and transfected with 0.2 µg plasmid DNA encoding mini-G_s_-NLuc (a gift from Prof. Nevin Lambert, Medical College of Georgia) (Wan et al., 2018), 0.2 µg KRAS-Venus or Rab5-Venus, plus 1.6 µg pcDNA3.1 for 24 hours before the assay. Cells were detached with EDTA and resuspended in HBSS with furimazine (1:50 dilution). After dispensing into 96-well white plates, a baseline read of luminescent signals at both 460 nm and 535 nm was serially recorded over 5 minutes using a Flexstation 3 instrument at 37°C. Ligands in HBSS were then added, after which signal was repeatedly recorded for 30 minutes. Results were first normalized to individual well baseline and then to the average vehicle signal from each experiment (Supplemental Fig. 5, G and H). Statistical comparisons were performed on AUC calculated from each ligand-induced kinetic trace.

### Insulin Secretion Assay

INS-1 832/3 cells were preincubated in complete medium at 3 mM glucose for 16 hours prior to the assay. After washing, cells were dispensed into 96-well plates containing 11 mM glucose ± the indicated concentration of agonist and incubated for 1 hour (acute secretion assay) or 16–18 hours (sustained secretion assay). Secreted insulin was analyzed from a sample of diluted supernatant by immunoassay (Cisbio wide range HTRF assay, measured using a Spectramax i3x plate reader). Results were normalized to the glucose-only response.

### Isolation and Imaging of Giant Unilamellar Vesicles

GUVs in the fluid phase, composed of 1-palmitoyl-2-oleoyl-sn-glycero-3-phosphocholine lipid were prepared via electroformation. Lipids were purchased from Avanti Polar Lipids. First, lipid in chloroform (20 µl; 1 mg/ml) was spread evenly on a conductive indium tin oxide–coated slide, leaving a film that was dried under vacuum for 30 minutes to remove residual solvent. A 5-mm-thick polydimethylsiloxane spacer with a central cutout was used to separate the slides with the conductive sides facing each other, and the chamber was filled with a solution of 100 mM sucrose in DI water. An alternating electric field (1 V, 10 Hz) was applied across the indium tin oxide–coated plates using a function generator (Aim-TTi, TG315). After 2 h, the electric field was changed to 1 V, 2 Hz for a further hour, and the resulting vesicles were collected. Either acylated or nonacylated compounds were added to make a final concentration of 100 nmol/l, and the solution was incubated for 30 minutes. For imaging, 10 μl of the vesicle suspension was added to 90 μl of 100 mM glucose in DI water in an imaging chamber, and the vesicles sedimented on the bottom of the glass slide. Confocal imaging was done on a Leica TCS SP5 confocal fluorescent microscope with a 20× objective set with an 84.5-µm pinhole (1 airy unit). Samples were acquired at a frequency of 400 Hz with eight line averages. The excitation was achieved at a wavelength of 495 nm, with emission set at between 510 and 530 nm. The images were acquired in the midplane of the GUVs. During data collection, focal planes slightly above and below were viewed to confirm image acquisition from the midplane.

### Peptide-Membrane Interaction Assay by FRET

T-REx-SNAP-GLP-1R cells were pretreated ± 0.1 µg/ml tetracycline to induce SNAP-GLP-1R expression. Cells were detached using EDTA and centrifuged, and the pellet was washed before the assay. Cellular labeling was then performed for 5 minutes using freshly prepared NR12S (Kucherak et al., 2010), a gift from Prof. Andrey Klymchenko, University of Strasbourg, at the indicated concentration in PBS in the dark. After washing three times to remove unbound probe, cells were dispensed into black 96-well microplates containing FITC-conjugated peptides at a final concentration of 100 nM. FRET measurements were taken using a Flexstation 3 instrument with the following settings: *λ*_ex_ 475 nm, *λ*_em_ range 520–680 nm in 5-nm increments, cutoff 495 nm. Recorded fluorescence intensities were then normalized to 525 nm to allow for well-to-well differences and signal quenching by FRET. The normalized spectral trace from wells containing cells without NR12S probe was subtracted to determine probe-related FRET signal, which was quantified as AUC between 525 and 680 nm. Gaussian fitting was performed to determine the peak emission wavelength and relative intensity.

### GLP-1R Conformational Sensor Assay

HEK293-SNAP-GLP-1R cells were labeled with Lumi4-Tb (40 nM, 60 minutes at 37°C, in complete medium). After washing, cells were resuspended in HBSS containing NR12S (50 nM) and seeded into half-area opaque white plates. Baseline signal was measured for 5 minutes at 37°C using a Flexstation 3 plate reader with the following settings: *λ*_ex_ = 335 nm, *λ*_em_ = 490 and 590 nm, delay 50 μs, integration time 300 μs. Ligands were then added, and signal was serially monitored for 10 minutes. The TR-FRET ratio, i.e., the ratio of fluorescence intensities at 590 and 490 nm, was considered indicative of the proximity of the GLP-1R extracellular domain (ECD) to the plasma membrane.

### Assessment of cAMP Signaling from Membrane Nanodomains by FRET

T-REx-SNAP-GLP-1R cells were transfected for 36 hours with 2 µg per well of a six-well plate of the cAMP biosensor AKAP79-CUTie (a gift from Prof. Manuela Zaccolo, University of Oxford) (Surdo et al., 2017). At 6 hours after transfection, cells were transferred to black, clear-bottom imaging microplates in complete medium containing 0.1 µg/ml tetracycline to induce SNAP-GLP-1R expression over 24 hours. For imaging experiments, adherent cells in HBSS underwent FRET imaging by widefield microscopy using an image splitter (Cairn Optosplit III) to separate cyan fluorescent protein- and yellow fluorescence protein-wavelength emission light into separate images on the camera chip. Images were acquired every second for a 1-minute baseline period and then for 5 minutes after addition of a saturating 1 µM agonist concentration. Channel registration was performed using the Cairn Image Splitter plug-in for ImageJ. Phase contrast images were used for segmentation, allowing quantification of mean fluorescence intensity from cell-containing and background fluorescence, with the latter being subtracted before calculation of cell-associated FRET ratio from each image. FRET signal was normalized to *t* = 0 and then to the vehicle response. For plate reader experiments, FRET signal was monitored from wells before and up to 12 minutes after addition of a range of concentrations of each agonist using a Flexstation 3 plate reader at 37°C and the following settings: *λ*_ex_ = 440 nm, *λ*_em_ = 485 and 535 nm. FRET was quantified as the ratio of fluorescent signal at 535 nm to that at 485 nm after subtraction of background signal at each wavelength. Cisbio lysis buffer was added immediately after completing the read, and total cAMP was determined by HTRF. Concentration-response curves were determined from average FRET signal from 8–12 minutes as well as the HTRF cAMP results to allow comparison of responses at an equivalent time point.

### Fluorescence Correlation Spectroscopy

Fluorescence correlation spectroscopy was performed as previously described (Corriden et al., 2014) on a Zeiss LSM 510NLO ConfoCor 3 microscope fitted with a 40× c-Apochromat 1.2 NA water-immersion objective. At 22°C, 30-second reads were taken with a diode-pumped solid-state 561-nm laser excitation at ∼0.2 kWcm^−2^, emission was collected through a 580- to 610-nm bandpass, and the pinhole was set to 1 airy unit. The measurement volume was calibrated on each experimental day with 20 nM Rhodamine 6G (diffusion coefficient, D, 2.8 × 10^−10^ m^2^s^−1^) in high performance liquid chromatography-grade water. Exendin-4-TMR and Exendin-4-TMR-C16 were prepared in HBSS with or without 0.1% BSA in an eight-well Nunc Laboratory-tek chambered coverglass (No. 1.0 borosilicate glass bottom) with the measurement volume placed 200 µm above the coverslip bottom. Autocorrelation curves were modeled in Zen 2012 (Carl Zeiss, Jena) to describe a single 3D diffusing component under free diffusion (Corriden et al., 2014). To determine the average molecular brightness of the labeled peptide, measurement reads were reanalyzed by photon counting histogram (PCH) analysis within Zen 2012 (Huang et al., 2004). PCH analysis can quantify the average molecular brightness (photon counts per molecule) of a species and also provides a means to separate and quantify the concentration of two species that differ in their average molecular brightness. The laser beam profile was approximated to follow a 3D Gaussian distribution, measurement reads were binned at 20 µs, appropriate for fluorescent species in solution, and the first order correction was calculated daily following calibration with 20 nM Rhodamine 6G.

### In Vivo Study

Animals were maintained in specific pathogen–free facilities, with ad libitum access to food (except prior to fasting studies) and water. Studies were regulated by the UK Animals (Scientific Procedures) Act 1986 of the UK and approved by Imperial College London (Project License PB7CFFE7A). Male C57Bl/6 mice (Charles River, UK) were fed a 60% high-fat diet (D12492; Research Diets) for 3 months prior to the study to induce obesity and glucose intolerance. The study began after an additional 1-week acclimatization period during which mice were singly housed and received sham intraperitoneal injections. Mice were randomly allocated to treatment, with average group weight confirmed to be similar postrandomization. The study began at the beginning of the dark phase with a single dose of each agonist or vehicle, combined with an intraperitoneal glucose tolerance test (IPGTT). Agonist was prepared in 20% glucose solution at a volume to provide the indicated weight-adjusted agonist dose and 2 g/kg glucose. Blood glucose was monitored before and at 20-minute intervals after agonist/vehicle/glucose administration using a handheld glucose meter. After 72 hours, the IPGTT was repeated at the beginning of the dark phase without further agonist administration. Food intake was assessed during the study by weighing of food at set intervals. Changes to body weight were also assessed at set intervals.

### Experimental Design and Statistical Analysis Considerations

A preliminary finding that exendin-4-C16 shows reduced *β*-arrestin-2 recruitment compared with exendin-4 (Lucey et al., 2020) gave us a reasonable expectation that the two ligands would display alterations to other aspects of their pharmacology. Therefore, we adopted an approach in which we planned to perform at least five independent replicates for quantitative cell culture assays as recommended by some authorities (Curtis et al., 2018). We did not perform a formal power calculation. In some instances, we observed more subtle differences between compounds that required additional repeats to gain confidence in the magnitude of the effects size, which were then performed to allow comparisons between assays evaluating similar phenomena (e.g., BRET vs. TR-FRET assays). Animal experiments were performed with seven to eight mice per group without a formal power calculation but on the basis of our prior experience in determining meaningful differences between GLP-1RAs. The investigators were not fully blinded for in vivo studies but did not have reference to treatment allocation while conducting experiments; in vitro experiments were unblinded. No mice were excluded from the analyses. In cell culture experiments, technical replicates were averaged so that each individual experiment was treated as one biologic replicate. Quantitative data were analyzed using Prism 8.0 (GraphPad Software). Dose responses were analyzed using three- or four-parameter logistic fits, with constraints imposed as appropriate. Bias analyses were performed as described in the section *NanoBiT Assay*. Statistical comparisons were made by *t* test or ANOVA as appropriate, with paired or matched designs used depending on the experimental design. Means ± S.E.M., with individual replicates in some cases, are displayed throughout. Statistical significance was inferred if *P* < 0.05, without ascribing additional levels of significance.

## Results

### Exendin-4-C16 Is a G Protein–Biased Ligand at GLP-1R

**Fig. 1.**
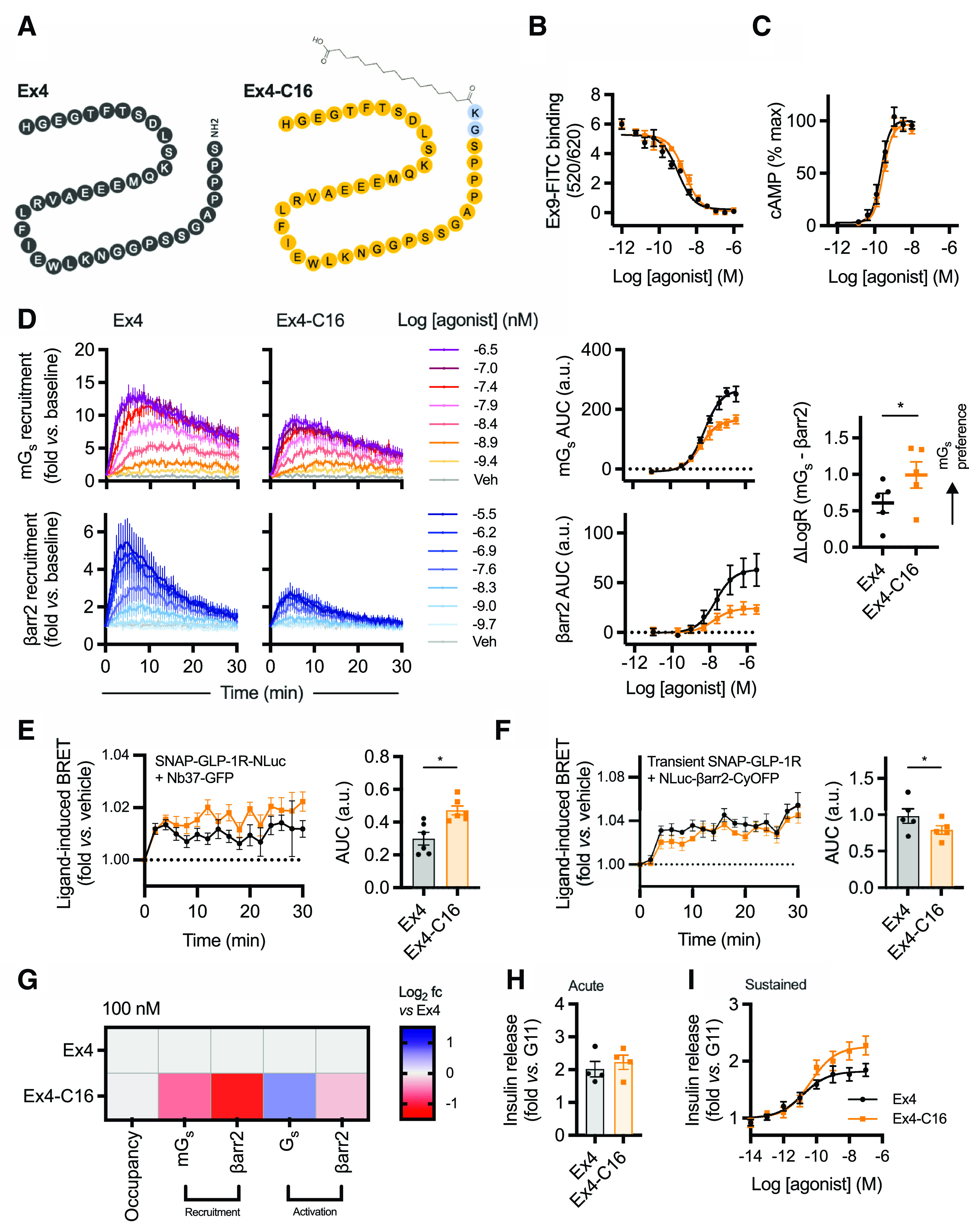
Biased agonism with exendin-4 and exendin-4-C16. (A) Schematic depicting the amino acid sequences of exendin-4 and exendin-4-C16 in single-letter amino acid code. (B) Equilibrium binding of unmodified exendin-4 and exendin-4-C16, *n* = 5, measured by competition binding in HEK293-SNAP-GLP1-R cells with exendin(9-39)-FITC used as the competing probe. (C) cAMP response in HEK293-SNAP-GLP-1R cells, 30-minute stimulation, *n* = 5, with four-parameter fits shown after normalization to global maximum. (D) LgBiT-mini-G_s_ (mG_s_) and LgBit-*β*-arrestin-2 (*β*arr2) recruitment to GLP-1R-SmBiT, *n* = 5, with three-parameter concentration-responses constructed from AUCs and bias factor calculation (see *Materials and Methods*) with comparison by paired *t* test. (E) Measurement of G*α*_s_ activation in HEK293T cells transiently transfected with SNAP-GLP-1R-NLuc and Nb37-GFP and stimulated with 100 nM agonist or vehicle, *n* = 6, with AUCs compared by paired *t* test. Refer to Supplemental Fig. 5B for individual experimental repeats. (F) Measurement of *β*-arrestin-2 activation in HEK293T cells transiently transfected with SNAP-GLP-1R and NLuc-4myc-*β*arr2-CYOFP1 and stimulated with 100 nM agonist or vehicle, *n* = 5, with AUCs compared by paired *t* test. (G) Heatmap representation of 100 nM agonist response data shown in Fig. 1, B–F with normalization to exendin-4 response. (H) Insulin secretion in INS-1 832/3 cells stimulated for 60 minutes with each agonist at 11 mM glucose (G11), *n* = 4, paired *t* test. (I) As for (H), but 16-hour stimulation, *n* = 6. Data are shown as means ± S.E.M. with individual replicates shown in some cases. *P < 0.05 by statistical test indicated.

An exendin-4 analog with a C16 diacid at the C terminus with a GK linker, originally described in our earlier publication (Lucey et al., 2020), was used in the present study and is referred to as “exendin-4-C16” (Fig. 1A). Competition binding experiments with FITC-conjugated antagonist ligand exendin(9-39) in HEK293 cells stably expressing SNAP-GLP-1R (Fang et al., 2020b) indicated an approximately 2-fold reduction in binding affinity for exendin-4-C16 compared with unmodified exendin-4 (Fig. 1B; Table 1). A minor reduction in cAMP signaling potency that was not statistically significant was also observed (Fig. 1C; Table 1). To investigate the possibility that exendin-4-C16 may display altered preference for coupling to intracellular effectors, i.e., biased agonism, we used nanoluciferase complementation to measure recruitment of mini-G_s_ (Wan et al., 2018) and *β*-arrestin-2 (Dixon et al., 2016) to the GLP-1R. This assay indicated that efficacy for *β*-arrestin-2 recruitment was particularly reduced with exendin-4-C16, with bias quantification using a standard approach (van der Westhuizen et al., 2014), confirming preferential coupling to mini-G_s_ recruitment (Fig. 1D; Table 1). *β*-Arrestin-1 and *β*-arrestin-2 recruitment were also measured using the PathHunter assay, showing no evidence of divergence in isoform selectivity (Supplemental Fig. 1A).

**TABLE 1:** Pharmacological parameters of exendin-4 and exendin-4-C16 Mean ± S.E.M. parameter estimates from concentration-response analyses in Fig. 1.

	Exendin-4	Exendin-4-C16
Binding affinity (HEK293-SNAP-GLP-1R, *n* = 5)		
Log K_i_ (M)	−9.2 ± 0.1	−8.8 ± 0.1*
Cyclic AMP (HEK293-SNAP-GLP-1R, *n* = 5)		
Log EC_50_ (M)	−9.7 ± 0.1	−9.5 ± 0.1
E_max_ (% global max)	101 ± 2	98 ± 3
Hill slope	2.1 ± 0.3	1.8 ± 0.3
Mini-G_s_ recruitment (HEK293T, *n* = 5)		
Log EC_50_ (M)	−8.2 ± 0.1	−8.3 ± 0.1
E_max_	268 ± 23	171 ± 14*
*β*-arrestin-2 recruitment (HEK293T, *n* = 5)		
Log EC_50_ (M)	−7.6 ± 0.1	−7.7 ± 0.1
E_max_ (AUC)	66 ± 16	25 ± 5*
Insulin secretion (INS-1 832/3, *n* = 6)		
Log EC_50_ (M)	−11.0 ± 0.2	−10.6 ± 0.1*
E_max_	1.7 ± 0.1	2.2 ± 0.2*

*P < 0.05 by paired *t* test.

As these complementation assays measure effector recruitment but not activation, we also performed further experiments to detect ligand-induced conformational changes in G_s_ and *β*-arrestin-2. For the former, we recorded dynamic changes in intermolecular BRET signal between GLP-1R tagged at the C terminus with nanoluciferase and GFP-tagged Nb37, a genetically encoded intrabody that recognizes and is recruited to active G*α*_s_ conformations (Irannejad et al., 2013). For the latter, we used an intramolecular *β*-arrestin-2 BRET sensor in which conformational changes lead to an increase in proximity between nanoluciferase at the N terminus and CyOFP at the C terminus (Oishi et al., 2019); this phenomenon is typically thought to indicate transition to an active state that could facilitate kinase activation (Nuber et al., 2016), although this was not specifically determined in our study. These studies suggested reduced *β*-arrestin-2 conformational rearrangement with exendin-4-C16 but, interestingly, increased G*α*_s_ activation (Fig. 1, E and F; Supplemental Fig. 1B), which could be a consequence of reduced *β*-arrestin–mediated desensitization. This G*α*_s_ activation assay showed a low dynamic range, but Ex4-C16 consistently showed a subtly increased response across all experimental repeats (Supplemental Fig. 5B). Note that the apparent increase in G*α*_s_ activation is still compatible with a lack of difference in measured E_max_ for cAMP (Fig. 1C), as adenylate cyclase activity may be saturated at submaximal activation of the cellular G*α*_s_ pool. Ligand pharmacology is summarized in the heatmap shown in Fig. 1G, indicating how, at a 100 nM dose, to achieve close to 100% receptor occupancy, G_s_ recruitment and activation are favored compared with *β*-arrestin-2 responses with exendin-4-C16.

As G protein–directed agonism is now established as a means to improve GLP-1R–mediated insulinotropic efficacy by avoiding receptor desensitization over prolonged stimulation periods (Jones et al., 2018; Fremaux et al., 2019; Fang et al., 2020b; Lucey et al., 2020), we also treated INS-1 832/3 clonal *β* cells (Hohmeier et al., 2000) for both 1 and 16 hours with exendin-4 and exendin-4-C16 and measured cumulative insulin secretion. As expected, there was no difference in acute insulin release (Fig. 1H), but the maximum response was increased with the -C16 ligand (Fig. 1I; Table 1).

### Exendin-4-C16 Triggers Slower GLP-1R Internalization Compared with Exendin-4

**Fig. 2.**
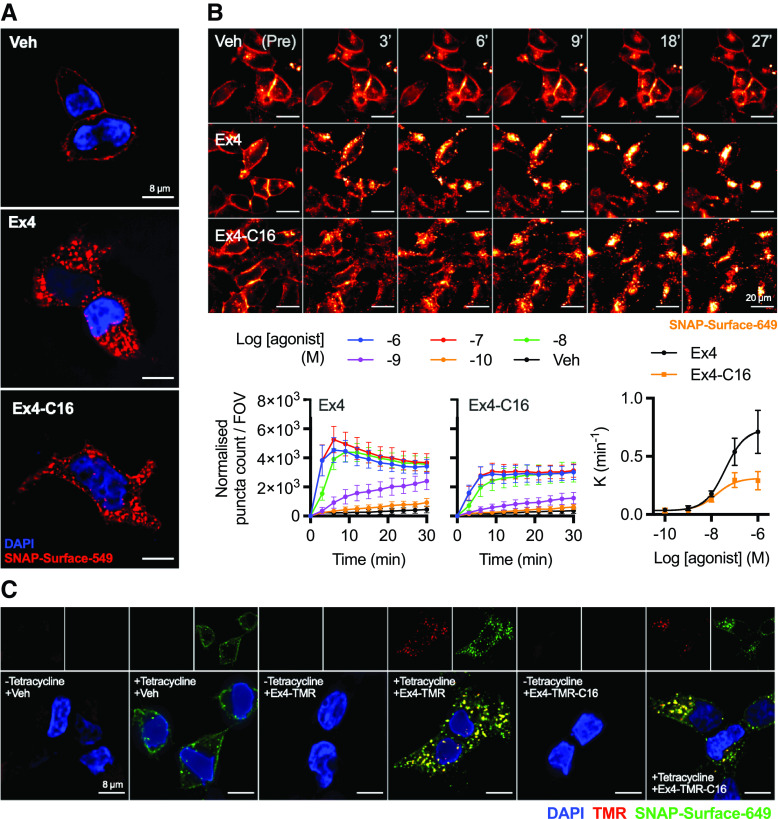
Visualization of GLP-1R endocytosis with exendin-4 and exendin-4-C16, and fluorescent agonist conjugates. (A) Representative images from *n* = 3 experiments demonstrating endocytosis of SNAP-GLP-1R labeled with SNAP-Surface-549 after treatment of 30 minutes with 100 nM agonist or vehicle; scale bars, 8 µm. (B) Time-lapse images demonstrating movement of surface-labeled SNAP-GLP-1R into punctate structures on stimulation with 100 nM agonist or vehicle; scale bars, 20 µm. The graphs show the change over time in “spot count” per 0.33-mm field of view (FOV) after normalization to cell confluence, with the concentration-dependent rate constant (K) derived from one-phase association fitting also plotted with a three-parameter fit. (C) Representative images showing endosomal uptake of exendin-4 and exendin-4-TMR in T-REx-SNAP-GLP-1R cells with or without tetracycline-induced GLP-1R expression, labeled with SNAP-Surface-649 prior to stimulation with 100 nM of each TMR-conjugate for 30 minutes. Scale bars, 8 µm. Data are shown as means ± S.E.M.

GLP-1R undergoes rapid agonist-induced internalization (Widmann et al., 1995), a process that is intrinsically linked to the spatial orchestration of intracellular signal generation (Fletcher et al., 2018; Tomas et al., 2019; Manchanda et al., 2021). Both exendin-4 and exendin-4-C16 resulted in extensive endocytosis of surface-labeled SNAP-GLP-1R (Fig. 2A). To quantify this process, we used high-content time-lapse microscopy in which the translocation of surface-labeled SNAP-GLP-1R into the endocytic network is determined from the appearance an intracellular punctate distribution of fluorescent signal (Fig. 2B; Supplemental Fig. 2A; Supplemental Video 1). Treatment with exendin-4 resulted in faster and more extensive appearance of fluorescent puncta at higher ligand concentrations. Notably, the number of puncta tended to reduce at later time points with exendin-4 because of the coalescence in a perinuclear location where multiple punctate endosomal structures which could no longer be individually resolved.

We also used tetramethylrhodamine (TMR)-tagged conjugates of each ligand, with the fluorophore installed at position K12, previously shown to be well tolerated by exendin-4 (Clardy et al., 2014; Jones et al., 2018; Pickford et al., 2020) and validated for GLP-1R binding by TR-FRET (Supplemental Fig. 2B) and cAMP measurements (Supplemental Fig. 2C), with the latter showing modest reductions in potency for both TMR conjugates. Using the tetracycline-inducible T-REx system to modulate SNAP-GLP-1R expression (Fang et al., 2020a), both fluorescent ligands were shown to bind specifically to the receptor and demonstrated extensive uptake into the endosomal compartment (Fig. 2C).

### Endosomal Trafficking Differences between Exendin-4 and Exendin-4-C16

**Fig. 3.**
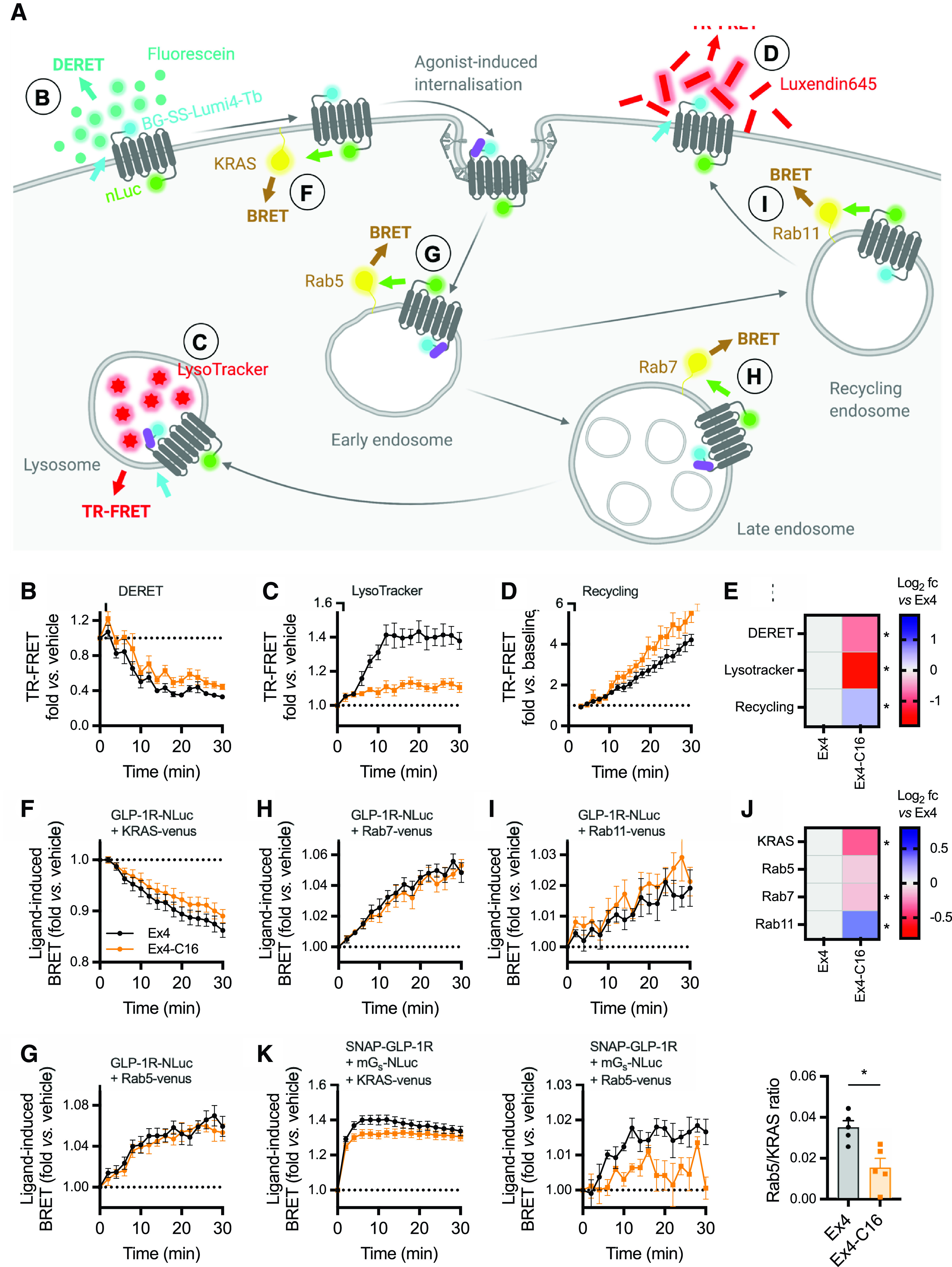
Subcellular targeting of exendin-4 and exendin-4-C16. (A) Schematic depicting the investigation of GLP-1R membrane trafficking using the TR-FRET and BRET assays used in this work. (B) GLP-1R disappearance from cell surface in HEK293-SNAP-GLP-1R cells treated with 100 nM agonist or vehicle, measured by DERET, *n* = 5. (C) GLP-1R translocation to late endosomes/lysosomes in HEK293-SNAP-GLP-1R cells treated with 100 nM agonist or vehicle, measured by TR-FRET using LysoTracker Red DND99 as the acceptor, *n* = 5. (D) GLP-1R recycling in HEK293-SNAP-GLP-1R cells previously treated for 30 minutes with 100 nM agonist, measured by TR-FRET using extracellular Luxendin645 as the acceptor, *n* = 5. (E) Heatmap representation of data shown in (B), (C), and (D) after normalization to the exendin-4 response; statistical significance is indicated after paired *t* test. (F) GLP-1R disappearance from the cell surface in HEK293T cells transiently transfected with SNAP-GLP-1R-NLuc and KRAS-venus and treated with 100 nM agonist or vehicle, measured by BRET, *n* = 6. (G) GLP-1R appearance in Rab5+ early endosomes in HEK293T cells transiently transfected with SNAP-GLP-1R-NLuc and KRAS-venus and treated with 100 nM agonist or vehicle, measured by BRET, *n* = 6. (H) As for (G), but for Rab7+ late endosomes, *n* = 7. (I) As for (G), but for Rab11+ recycling endosomes, *n* = 7. (J) Heatmap representation of data shown in (F), (G), (H), and (I) after normalization to the exendin-4 response; statistical significance is indicated from paired *t* tests. (K) Mini-G_s_ translocation to the plasma membrane or Rab5+ early endosomes in HEK293-SNAP-GLP-1R cells treated with 100 nM agonist after transient transfection with Mini-G_s_-NLuc plus KRAS-venus or Rab5-venus, *n* = 5. The agonist-specific AUC measured for each compartment marker are ratiometrically compared by paired *t* test. Data are shown as means ± S.E.M. with individual replicates shown in some cases. *P < 0.05 by statistical test indicated.

To gain insights into ligand-induced GLP-1R movements to or from different subcellular compartments, we used time-resolved proximity-based energy transfer techniques, with both chemical (for FRET) or genetically encoded (for BRET) acceptors as localization markers, providing complementary readouts. SNAP-GLP-1R labeled at the N terminus with the lanthanide probe Lumi4-Tb can transfer energy to time-resolved FRET acceptors situated in the extracellular space or the endosomal lumen (Fig. 3A). DERET was used to quantify movement of GLP-1R away from the cell surface as a reduction in signal transfer to fluorescein-containing extracellular buffer (Levoye et al., 2015); this confirmed that GLP-1R internalization was slower for exendin-4-C16 (Fig. 3B; Supplemental Fig. 3A). We developed a further assay in which the trafficking of GLP-1R to late endosomes/lysosomes was detected by energy transfer to the lysomotropic fluorescent dye LysoTracker-DND99 (Fig. 3C). This suggested that exendin-4-C16 treatment led to markedly less targeting of GLP-1R to this degradative compartment and, as this difference was more marked than for the internalization reading by DERET, that this reflects a difference in postendocytic targeting rather than simply less GLP-1R internalization. In contrast, postendocytic recycling of GLP-1R back to the plasma membrane, measured by time-resolved FRET (Fang et al., 2020b) between reemergent GLP-1R and Cy5-labeled antagonist analog of exendin(9-39) (Ast et al., 2020), was faster after treatment with exendin-4-C16 (Fig. 3D). TR-FRET data are also summarized and statistically compared in Fig. 3E.

Bystander BRET can be used to monitor the translocation of GPCRs within the endocytic network (Tiulpakov et al., 2016). We observed a ligand-induced reduction in BRET signal when SNAP-GLP-1R tagged at the C terminus with nanoluciferase was coexpressed with the plasma membrane marker KRAS-Venus; the effect was more pronounced with exendin-4 than with exendin-4-C16 (Fig. 3F). A concomitant increase in BRET signal to Rab5-Venus was seen, indicating entry of the receptor into early endosomes (Fig. 3G). The similar Rab5 BRET signal for each ligand in the face of apparently different GLP-1R internalization rates might be reconciled by the observation that, after exendin-4-C16 treatment, a subtly reduced signal from Rab7-positive late endosomes was detected (Fig. 3H), whereas a greater signal was recorded from Rab11-positive recycling endosomes (Fig. 3I). This pattern is suggestive of preferential trafficking of GLP-1R toward a recycling pathway with exendin-4-C16 treatment, resulting in a reduced net rate of surface receptor loss. BRET results were broadly in accordance with results from the TR-FRET assays, although differences were generally smaller while remaining statistically significant (Fig. 3J).

GLP-1R cAMP signaling was reported to originate from early endosomes as well as the plasma membrane (Girada et al., 2017). As an indirect readout of this process, we monitored the redistribution of nanoluciferase-tagged mini-G_s_ to different subcellular compartments after agonist stimulation. A rapid increase in mini-G_s_-to-KRAS (plasma membrane) BRET signal was apparent with both ligands (Fig. 3K), but with a slightly higher peak response with exendin-4, consistent with the higher efficacy displayed by this ligand for G protein recruitment (Fig. 1D). A decline in signal at later time points was observed with exendin-4 but not exendin-4-C16, which may result from more extensive *β*-arrestin–mediated steric hinderance or internalization of GLP-1R with the former ligand. Interestingly, mini-G_s_-to-Rab5 (early endosome) BRET signal amplitude was considerably reduced for exendin-4-C16 (Fig. 3K), despite the fact that the receptor localization in the Rab5 compartment was similar for both ligands (Fig. 3G).

These data indicate that C-terminal acylation alters the trafficking profile of exendin-4, favoring GLP-1R sorting toward a recycling rather than degradative pathway. The observation that mini-G_s_ protein recruitment to early endosomes was reduced with exendin-4-C16, yet this ligand shows higher efficacy for sustained insulin secretion, suggests that signaling from early endosomes may not be a dominant mechanism for prolonged signaling with GLP-1R under pharmacological conditions, at least with exendin-4 derived GLP-1R agonists.

### Acylation Affects the Interaction of the Exendin-4 Peptide with the Plasma Membrane

**Fig. 4.**
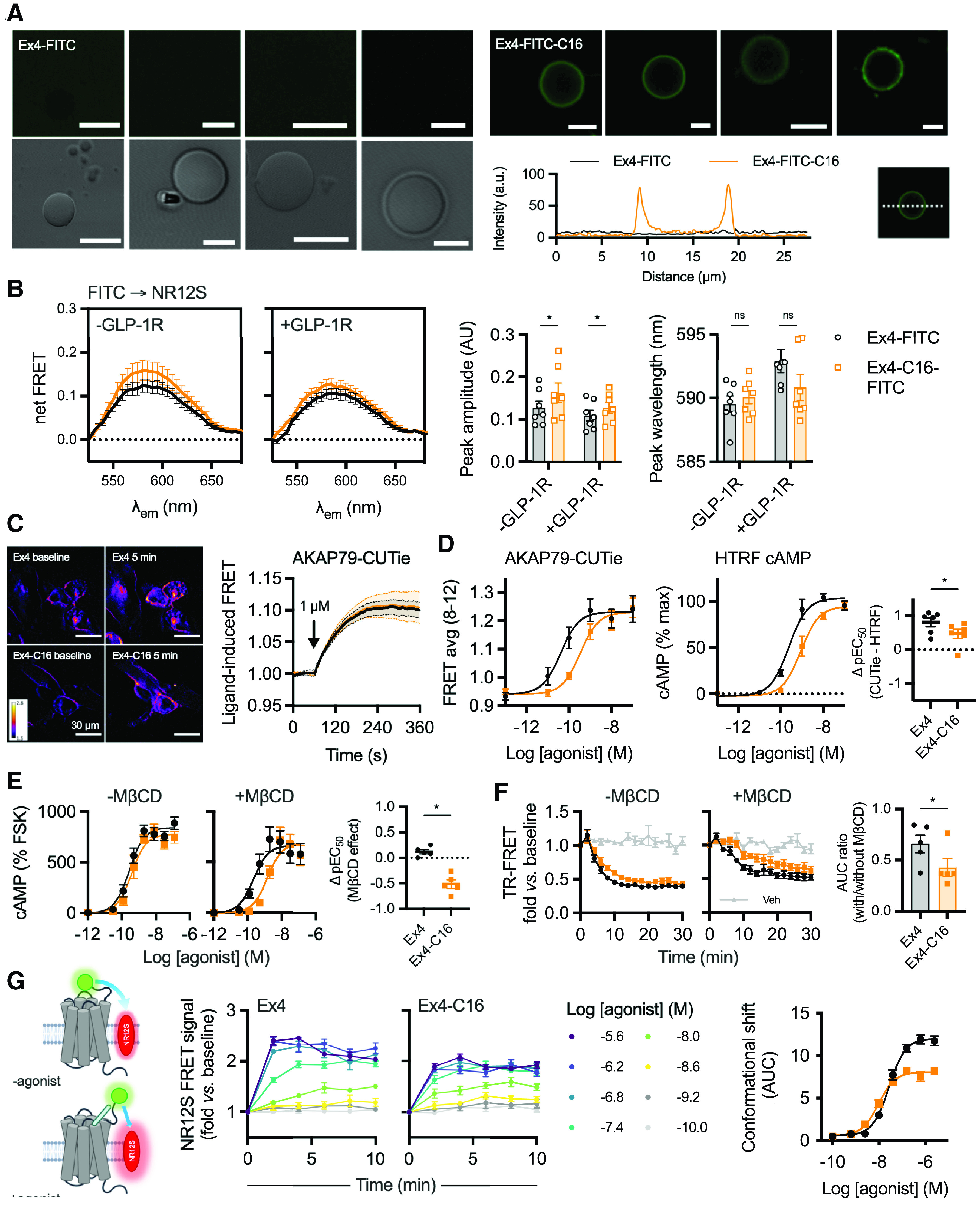
Membrane interactions of C-terminally acylated exendin-4 conjugates. (A) Confocal microscopy and phase contrast images of GUVs incubated with exendin-4-FITC or exendin-4-FITC-C16. Scale bars, 5 µm. Membrane signal is represented on the line plot. (B) FRET spectrum from T-REx-SNAP-GLP-1R cells with or without tetracycline-induced GLP-1R expression, labeled with 50 nM NR12S and incubated with 100 nM exendin-4-FITC or exendin-4-FITC-C16, *n* = 7. The background spectrum of each FITC ligand was subtracted, and the trace was normalized to the signal at 525 nm. Peak emission wavelength and amplitude, derived from Gaussian fitting of each spectrum, are shown and compared between ligands by two-way randomized block ANOVA with Sidak’s test. (C) FRET imaging of T-REx-SNAP-GLP-1R cells stimulated with 1 µM exendin-4 or exendin-4-C16, with representative ratiometric images pre- and poststimulation, and quantification from *n* =3 experiments. (D) Matched comparison of nanodomain-specific cAMP (AKAP79-CUTie) and total cAMP (HTRF) in T-REx-SNAP-GLP-1R cells, 12-minute stimulation, *n* = 6, with ΔpEC_50_ comparison by paired *t* test. (E) cAMP responses in HEK293-SNAP-GLP-1R cells preincubated for 30 minutes with vehicle or 10 mM M*β*CD prior to stimulation with each ligand for 30 minutes. Responses are expressed relative to the forskolin (FSK; 10 µM) response, *n* = 5, with the effect of M*β*CD on potency shown by subtracting pEC_50_ results and comparison by paired *t* test. (F) GLP-1R internalization measured by DERET in HEK293-SNAP-GLP-1R cells preincubated for 30 minutes with vehicle or 10 mM M*β*CD prior to stimulation with each ligand at 100 nM for 30 minutes, *n* = 5. AUC relative to baseline was quantified, and +M*β*CD results are expressed relative to −M*β*CD results for each ligand, and compared by paired *t* test. (G) Principle of NR12S conformational biosensor assay, with kinetic responses showing TR-FRET ratio normalized to baseline in response to indicated concentration of exendin-4 or exendin-4-C16, *n* = 5, with concentration-response curve constructed from AUC with three-parameter fit. Data are shown as means ± S.E.M. with individual replicates when possible. *P < 0.05 by statistical test indicated.

Several studies report an interaction of GLP-1R agonist peptides with model membranes, typically resulting in enhanced stability of the helical secondary structure (Thornton and Gorenstein, 1994; Neidigh et al., 2001; Andersen et al., 2002; Fox et al., 2009). Acylation of glucagon-like peptide-2 increases its tendency to interact with lipid bilayers (Trier et al., 2014). Similarly, the C16 chain of exendin-4-C16 might promote selective engagement with membrane regions dependent on local hydrophobicity. We performed confocal microscopy of FITC conjugates of exendin-4 and exendin-4-C16 bound to GUVs, prepared via electroformation. Both FITC ligands were conjugated at position K12, as for the equivalent TMR conjugates, and retained GLP-1R binding properties (Supplemental Fig. 4A) and cAMP signaling (Supplemental Fig. 4B). When the acylated compound was present in the sample, clear fluorescence accumulation was seen on the membrane surface, in contrast to the nonacylated compound, in which no accumulation was seen (Fig. 4A). This demonstrates that the presence of the C16 acyl chain promotes insertion of the molecule into model lipid bilayers.

To establish whether this phenomenon is relevant to living cells, we measured FRET between FITC ligands and the plasma membrane of T-REx-SNAP-GLP-1R cells labeled with the solvatochromic probe NR12S (Kucherak et al., 2010). The excitation spectrum of NR12S is well matched to the emission spectrum of FITC, and its emission spectrum is polarity-sensitive, potentially allowing discrimination of interactions occurring in membrane domains with different degrees of liquid order, e.g., lipid rafts versus nonraft regions. The NR12S spectra obtained from excitation of FITC ligands incubated with NR12S-labeled cells showed an increase in maximum FRET signal without any significant spectral shift (Fig. 4B). Similar findings were observed with or without GLP-1R expression in the same cell system (Fig. 4B). This suggests that exendin-4-C16 may indeed interact with cell membranes to a greater extent than does exendin-4 but does not provide evidence that this directs the ligand to interact with specific GLP-1R subpopulations situated in membrane nanodomains with different degrees of liquid order.

We also investigated the possibility of ligand-specific GLP-1R cAMP signal localization using the targeted cAMP FRET biosensor AKAP79-CUTie (Surdo et al., 2017). AKAP79 is a protein kinase A anchoring protein that is typically situated in membrane rafts by virtue of its palmitoylation (Delint-Ramirez et al., 2011). FRET imaging indicated similar kinetics for nanodomain-specific cAMP production with a saturating concentration of exendin-4 and exendin-4-C16 (Fig. 4C). Comparisons of ligand potencies for AKAP79-CUTie versus total cAMP measured by HTRF indicated that exendin-4–induced cAMP signaling is more constrained to AKAP79-marked nanodomains than for exendin-4-C16 (Fig. 4D; Supplemental Fig. 4C). On the other hand, pretreatment with methyl-*β*-cyclodextrin (M*β*CD) to sequester cholesterol and disrupt membrane nanodomain structure (Mahammad and Parmryd, 2015) disproportionately reduced cAMP production by exendin-4-C16 compared with exendin-4 (Fig. 4E). This might result from the lower G protein recruitment efficacy of exendin-4-C16, meaning that its ability to generate cAMP signals is more susceptible to uncoupling of G proteins from receptors as a result of disruption of membrane structure (Mystek et al., 2016). Similarly, exendin-4-C16–induced GLP-1R endocytosis, measured by TR-FRET, was more substantially affected by M*β*CD treatment than exendin-4 (Fig. 4F).

To gain further insights into how exendin-4 and exendin-4-C16 interact with GLP-1R at the plasma membrane, we used NR12S as a FRET acceptor for the N-terminally SNAP-tagged GLP-1R labeled with Lumi-4-Tb, monitoring dynamic changes in TR-FRET signal with each ligand over time indicative of movement of the receptor ECD relative to the plasma membrane (Fig. 4G). A similar principle has been used previously to monitor epidermal growth factor receptor conformational shifts by FRET microscopy (Ziomkiewicz et al., 2013). Here, both ligands led to clear increases in TR-FRET signal across a wide concentration range. Efficacy was reduced with exendin-4-C16 (Fig. 4G), in keeping with lower efficacy measurements for intracellular responses including recruitment of mini-G_s_ and *β*-arrestin-2 (see Fig. 1), although an increase in potency was observed. The implications of these responses are not clear, as they could reflect differences in ECD movement, known to be a feature of GLP-1R activation as determined from structural and computational studies (Zhang et al., 2019; Wu et al., 2020), but ligand-induced effects on membrane architecture cannot be ruled out.

### Interaction of Exendin-4-C16 with Albumin and In Vivo Efficacy

**Fig. 5.**
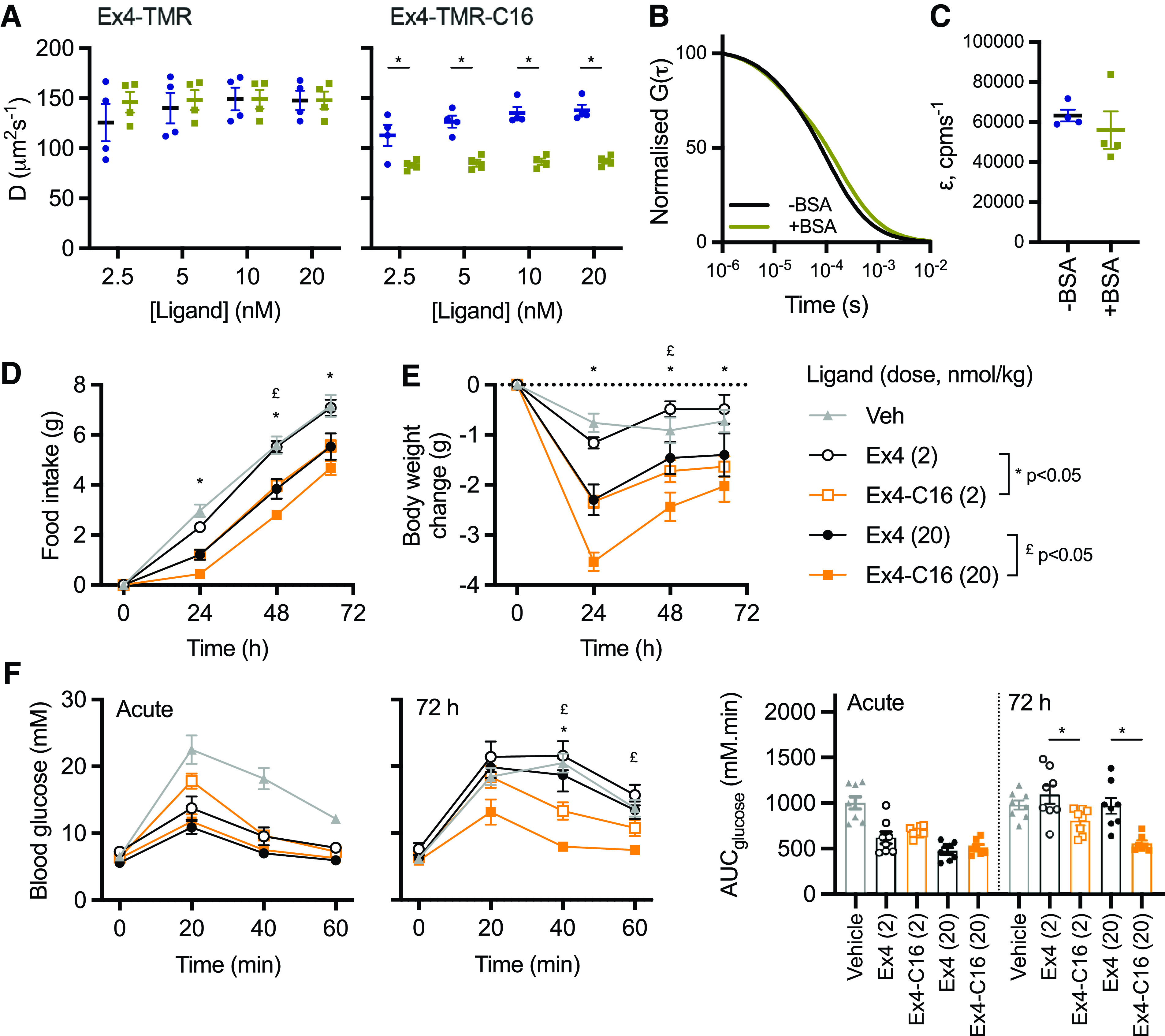
Albumin binding by fluorescence correlation spectroscopy and in vivo effects. (A) Diffusion coefficients, D (µm^2^s^−1^), of indicated concentration of indicated ligand with and without 0.1% BSA, with comparison by two-way randomized block ANOVA with Sidak’s test. (B) Representative 1x3D free diffusion fit for Exendin4-TMR-C16 (20 nM) autocorrelation curves with or without 0.1% BSA. The autocorrelation function, G(*τ*), is normalized to facilitate comparison of decay curves. (C) Molecular brightness, *ε* (counts per molecule per second), of EX4-TMR-C16 (20 nM) with and without 0.1% BSA. (D) Cumulative food intake in male HFD mice (*n* = 8/group) after a single intraperitoneal injection of the indicated agonist. Statistical comparisons between equimolar agonist doses by two-way repeated measures ANOVA with Tukey’s test. (E) As for (B), but showing the effect on body weight. (F) IPGTT (2 g/kg glucose) performed at the start and end of the 72-hour study. AUCs are compared by one-way ANOVA with Sidak’s test for equimolar agonist doses. Data are shown as means ± S.E.M. with individual replicates shown in some cases. *P < 0.05 by statistical test indicated.

The classic benefit of peptide acylation is to promote reversible binding to albumin in the circulation, thereby avoiding renal elimination. We previously showed that exendin-4-C16 and related analogs are >97% bound to proteins in human and mouse plasma (Lucey et al., 2020). In the present study, we performed fluorescence correlation spectroscopy to measure the interaction of both TMR-conjugated peptides with BSA (Fig. 5, A–C). All traces could be fitted to a 1x3D free diffusion model, indicating there is a single diffusing component. Diffusion coefficients for exendin-4-TMR and exendin-4-TMR-C16, tested at a range of concentrations between 2.5 and 20 nM, were similar in the absence of BSA (Fig. 5B). However, diffusion of exendin-4-TMR-C16 was markedly slowed in the presence of BSA, indicative of formation of peptide-albumin complexes. (Fig. 5, A and B). Exendin-4-TMR-C16 measurement reads were reanalyzed by PCH analysis with all reads fitting to a single-species PCH fit and the reported average molecular brightness unaffected by the addition of BSA (Fig. 5C). These results support our interpretation of a slowing of diffusion coefficient as a result of exendin-4-TMR-C16 interaction with BSA rather than an accumulation of peptide, which would have resulted in an increase in average molecular brightness.

Finally, the metabolic effects of exendin-4 and exendin-4-C16 were compared in mice in which glucose intolerance was first induced by high-fat feeding for 2 months prior to the study. Exendin-4-C16 was previously shown to be detectable in plasma 72 hours after dosing (Lucey et al., 2020). Therefore, the effect of a single administration of each ligand, at two separate doses, was assessed. Both doses of exendin-4-C16 led to greater suppression of food intake and weight loss throughout the dosing period (Fig. 5, D and E). Moreover, both doses of the acylated ligand exerted a larger antihyperglycemic effect in an intraperitoneal glucose tolerance test performed 72 hours after dosing (Fig. 5F).

## Discussion

This study demonstrates how C-terminal acylation of exendin-4 affects several GLP-1R pharmacological properties that are relevant to its therapeutic effect. Although exendin-4-C16 showed minimal reduction in cAMP signaling potency, marked differences were observed for recruitment of key intracellular effectors and trafficking responses, which led to increased insulin secretion with prolonged incubations. We also evaluated the effect of peptide acylation on binding to membranes and albumin to more comprehensively describe the differences between these ligands. Overall, our study highlights the breadth of pharmacological parameters than can be affected by peptide acylation.

This work was prompted by our earlier observation that C-terminal acylation of biased GLP-1RAs based on exendin-4 results in reduced recruitment efficacy for *β*-arrestin and mini-G protein (Lucey et al., 2020). The present study confirms this observation, with a 60% reduction in maximum response recorded for *β*-arrestin-2 recruitment by nanoBiT complementation with exendin-4-C16. Although mini-G_s_ recruitment was also reduced, the impact on *β*-arrestin-2 recruitment was greater, with biased agonism confirmed by the operational model approach and on the basis of efficacy differences (Onaran et al., 2017). We note that this does not indicate that the ligands show opposing preference for G protein– versus *β*-arrestin–favoring GLP-1R conformations; the observed bias more likely results from the fact that *β*-arrestin responses appear to be more susceptible to reductions in GLP-1R efficacy than do G protein responses. Indeed, our data add to the growing body of evidence that G protein–favoring biased GLP-1RAs typically show reduced efficacy for recruitment of both G proteins and *β*-arrestins (Fang et al., 2020b; Lucey et al., 2020; Pickford et al., 2020). However, using a novel Nb37-based BRET approach to monitor activation of endogenous G proteins close to GLP-1R, we demonstrate here that exendin-4-C16 shows increases in G*α*_s_ activation in spite of its lower mini-G_s_ recruitment efficacy. This could reflect an inherent difference in ability of exendin-4-C16 to activate G*α*_s_ in spite of reduced recruitment. However, we suspect the real reason is that the activation assay is more susceptible to normal regulatory processes, such as steric hindrance by *β*-arrestin recruitment, which fail to displace mini-G responses due to the highly stable nature of the GPCR-mini-G complexes (Carpenter and Tate, 2016). Although the dynamic range of this assay was low in our hands, making it challenging to apply to higher throughput screening efforts or to concentration responses, it could be adapted by the use of alternative fluorophores with greater signal separation from the nanoluciferase emission peak (Dale et al., 2019) or using complementation approaches (Inoue et al., 2019). Other approaches to monitor G protein activation have typically required overexpression of tagged G protein subunits (Masuho et al., 2015; Inoue et al., 2019; Olsen et al., 2020; Zhao et al., 2020), which may not totally replicate the physiologic setting, although corroboration using these approaches would be useful for validation.

In line with other studies showing that lower efficacy biased GLP-1RAs tend to induce slower GLP-1R endocytosis (Jones et al., 2018; Fremaux et al., 2019; Lucey et al., 2020; Willard et al., 2020), we observed that GLP-1R internalization was reduced with exendin-4-C16 compared with exendin-4. The automated microscopy approach we used to demonstrate this has certain advantages over lower throughput methods by allowing responses to a wide range of ligands or, in this case, ligand concentrations to be monitored in parallel across several fields of view, characterizing ligand effects in more detail and with greater statistical robustness. However, this method is unable to discriminate between GLP-1R clustering at the plasma membrane versus bona fide endocytosis events, although these are intrinsically linked, with the former occurring rapidly after ligand stimulation as a precursor to uptake into clathrin-coated vesicles (Buenaventura et al., 2019). The system could be adapted for use with alternative fluorescence approaches to monitor internalization, e.g., using pH-sensitive SNAP-labeling fluorophores (Martineau et al., 2017).

We also applied a series of complementary proximity-based techniques based on both BRET and FRET to monitor GLP-1R redistribution between the plasma membrane and different endosomal compartments. Monitoring GLP-1R disappearance from the plasma membrane by DERET has been widely applied (Roed et al., 2014; Jones et al., 2018), and the use of the cleavable BG-Lumi4-Tb to monitor GLP-1R recycling in combination with a fluorescent antagonist ligand was recently described by our group (Pickford et al., 2020). We extended this approach here to detect GLP-1R translocation to late endosomes and lysosomes marked by the lysomotropic dye LysoTracker, facilitated by the spectral overlap of one of the Tb emission peaks with the excitation spectrum of LysoTracker DND99. In principle, a similar approach could be trialed with other fluorescent markers that accumulate in different subcellular compartments. Using a nanoluciferase tag at the GLP-1R C terminus, we were also able to obtain BRET measurements of GLP-1R redistribution to early, late, and recycling endosomes that corroborate the TR-FRET responses. This approach has been used recently to study the trafficking profiles of GLP-1R monoagonists and dual GLP-1R/GIPR coagonists (Fletcher et al., 2018; Novikoff et al., 2021), albeit using *Renilla* luciferase rather than the nanoluciferase we used in our study. The rank order of ligand-induced changes was consistent for “matched” TR-FRET and BRET approaches, with exendin-4-C16 showing reduced GLP-1R internalization and lysosomal accumulation but faster recycling. In our hands. the TR-FRET approach resulted in clearer discrimination between ligand responses, which could reflect cell model differences, influence of the C-terminal NLuc tag for BRET, or assay/instrument sensitivity.

Importantly, we also observed differences in mini-G_s_ recruitment to plasma membrane versus early endosomes using the nanoBRET approach, providing some insight into compartmentalization of GLP1-R signaling. Here, plasma membrane mini-G_s_ recruitment was somewhat reduced for exendin-4-C16 compared with exendin-4, which is compatible with results from our nanoBiT complementation assay demonstrating reduced global mini-G_s_ recruitment efficacy for this ligand. However, recruitment of mini-G_s_ to Rab5-positive early endosomes was reduced to an even greater extent, which can be at least partly explained by the reduced rate of internalization with this ligand, although differences in ability to maintain GLP-1R activation once internalized could also contribute. Indeed, the GLP-1R-Rab5 BRET signal was only marginally reduced with exendin-4-C16 versus exendin-4, whereas the mini-G_s_-Rab5 BRET response showed larger differences between ligands. However, differences in luciferase/fluorophore configuration between assays, as well as unknown effects of mini-G_s_ overexpression on GLP-1R pharmacology, mean that further work will be needed to fully explain this observation. GLP-1R has been reported to generate signals from the endosomal compartment (Kuna et al., 2013; Roed et al., 2015; Girada et al., 2017; Fletcher et al., 2018), in line with many other GPCRs (Vilardaga et al., 2014), and this phenomenon is frequently claimed to be a mechanism for sustained cAMP signaling. However, although our results corroborate the existence of GLP-1R–associated endosomal signaling, they also suggest that sustained GLP-1R signaling, as indicated by cumulative insulin secretion over pharmacologically relevant timescales, is actually greater with the ligand (exendin-4-C16) with a reduced tendency to recruit mini-G_s_ to Rab5-positive endosomes. This raises questions about the relative therapeutic importance of maintaining an adequate pool of surface GLP-1Rs during prolonged stimulations versus aiming for maximal endosomal receptor activation.

The structural basis for the modified GLP-1R activation profile with exendin-4-C16 is not clear. The C terminus of exendin-4, although not required for GLP-1R activation (Lee et al., 2018), plays an important role in GLP-1R binding (Doyle et al., 2003). Installation of a large acyl chain at the peptide C terminus could potentially interfere with ligand binding. We observed a modest reduction in GLP-1R binding affinity, in keeping with this possibility. However, receptor activation efficacy was also reduced. Interestingly, the C terminus of exendin-4 may be required to facilitate formation of high-order GLP-1R oligomers through interaction with neighboring GLP-1R protomers in *trans* (Koole et al., 2017). GLP-1R oligomerization is reported to be required for full signaling responses (Harikumar et al., 2012). A further possibility is that the acyl chain could interact with the plasma membrane in a specific manner that interferes with GLP-1R activation. We found here that a fluorescently labeled exendin-4-C16 does indeed form interactions with model membranes, whereas the equivalently labeled nonacylated exendin-4 does not. Corresponding measurements from living cells also suggested greater membrane interactions with the acylated ligand but did not support the possibility of localized activation of GLP-1R subpopulations situated in particular plasma membrane nanodomains.

As differential ability of ligands to stabilize active GLP-1R conformations is a further potential explanation for the ligand signaling efficacy differences, we devised a strategy to monitor movements between the receptor ECD and the plasma membrane, finding efficacy reductions with exendin-4-C16 that matched the reduced intracellular signaling responses also observed with this ligand. This approach may be more widely useful as a conformational sensor for other GPCRs, although it lacks the ability to detect changes at the receptor intracellular face that is needed to provide insights into conformational changes required for G protein interactions.

There are a number of limitations with our study. Firstly, we investigated the effects of a single type of C16 diacid acyl chain, and our results are not necessarily extrapolable to GLP-1R agonists with different acyl chain lengths or acyl monoacids. Secondly, the majority of studies were performed in heterologous cell lines with overexpression of tagged GLP-1R constructs, which could influence the pharmacology. Although numerous studies have demonstrated that biased agonism and trafficking assessments in heterologous systems reliably predict insulin secretory and other key physiologic effects of biased GLP-1RAs in vivo, the field would benefit from further efforts to study behaviors of endogenous GLP-1R in *β* cells and cell types with native GLP-1R expression.

In summary, beyond the expected effects on binding to albumin, C-terminal acylation of exendin-4 led to changes in multiple pharmacological parameters relevant to downstream GLP-1R responses. These observations are more broadly relevant to drug discovery at peptide GPCRs for which ligand acylation is a valid approach to improve pharmacokinetics.

## Abbreviations

3D: 3 dimensional
AUC: area under curve
BRET: bioluminescence resonance energy transfer
BSA: bovine serum albumin
CyOFP: cyan-excitable orange fluorescent protein
DERET: diffusion-enhanced resonance energy transfer
DMEM: Dulbecco’s modified Eagle’s medium
ECD: extracellular domain
FITC: fluorescein isothiocyanate
FRET: forster resonance energy transfer
GLP: glucagon-like peptide
GLP-1R: glucagon-like peptide-1 receptor
GLP-1RA: glucagon-like peptide-1 receptor agonist
GPCR: G protein–coupled receptor
GUV: giant unilameller vesicle
HBSS: Hanks’ balanced salt solution
HTRF: homogeneous time-resolved fluorescence
IPGTT: intraperitoneal glucose tolerance test
M*β*CD: methyl-*β*-cyclodextrin
NA: numerical apperture
Nb37: nanobody-37
PCH: photon counting histogram
Tb: terbium
T2D: type 2 diabetes
TMR: tetramethylrhodamine
TR-FRET: time-resolved Förster resonance energy transfer

## References

[B1] Andersen A Lund A Knop FK Vilsbøll T (2018) Glucagon-like peptide 1 in health and disease. Nat Rev Endocrinol 14:390–403. 29728598 10.1038/s41574-018-0016-2

[B2] Andersen NH Brodsky Y Neidigh JW Prickett KS (2002) Medium-dependence of the secondary structure of exendin-4 and glucagon-like-peptide-1. Bioorg Med Chem 10:79–85. 11738609 10.1016/s0968-0896(01)00263-2

[B3] Ast J Arvaniti A Fine NHF Nasteska D Ashford FB Stamataki Z Koszegi Z Bacon A Jones BJ Lucey MA, (2020) Super-resolution microscopy compatible fluorescent probes reveal endogenous glucagon-like peptide-1 receptor distribution and dynamics. Nat Commun 11:467. 31980626 10.1038/s41467-020-14309-w PMC6981144

[B4] Buenaventura T Bitsi S Laughlin WE Burgoyne T Lyu Z Oqua AI Norman H McGlone ER Klymchenko AS Corrêa IR Jr, (2019) Agonist-induced membrane nanodomain clustering drives GLP-1 receptor responses in pancreatic beta cells. PLoS Biol 17:e3000097. 31430273 10.1371/journal.pbio.3000097 PMC6716783

[B5] Carpenter B Tate CG (2016) Engineering a minimal G protein to facilitate crystallisation of G protein-coupled receptors in their active conformation. Protein Eng Des Sel 29:583–594. 27672048 10.1093/protein/gzw049 PMC5181381

[B6] Clardy SM Keliher EJ Mohan JF Sebas M Benoist C Mathis D Weissleder R (2014) Fluorescent exendin-4 derivatives for pancreatic β-cell analysis. Bioconjug Chem 25:171–177. 24328216 10.1021/bc4005014 PMC4016126

[B7] Corriden R Kilpatrick LE Kellam B Briddon SJ Hill SJ (2014) Kinetic analysis of antagonist-occupied adenosine-A3 receptors within membrane microdomains of individual cells provides evidence of receptor dimerization and allosterism. FASEB J 28:4211–4222. 24970394 10.1096/fj.13-247270 PMC4202110

[B8] Curtis MJ Alexander S Cirino G Docherty JR George CH Giembycz MA Hoyer D Insel PA Izzo AA Ji Y, (2018) Experimental design and analysis and their reporting II: updated and simplified guidance for authors and peer reviewers. Br J Pharmacol 175:987–993. 29520785 10.1111/bph.14153 PMC5843711

[B9] Dale NC Johnstone EKM White CW Pfleger KDG (2019) NanoBRET: the bright future of proximity-based assays. Front Bioeng Biotechnol 7:56. 30972335 10.3389/fbioe.2019.00056 PMC6443706

[B10] Delint-Ramirez I Willoughby D Hammond GRV Ayling LJ Cooper DM Cooper DMF (2011) Palmitoylation targets AKAP79 protein to lipid rafts and promotes its regulation of calcium-sensitive adenylyl cyclase type 8. J Biol Chem 286:32962–32975. 21771783 10.1074/jbc.M111.243899 PMC3190942

[B11] Dixon AS Schwinn MK Hall MP Zimmerman K Otto P Lubben TH Butler BL Binkowski BF Machleidt T Kirkland TA, (2016) NanoLuc complementation reporter optimized for accurate measurement of protein interactions in cells. ACS Chem Biol 11:400–408. 26569370 10.1021/acschembio.5b00753

[B12] Doyle ME Theodorakis MJ Holloway HW Bernier M Greig NH Egan JM (2003) The importance of the nine-amino acid C-terminal sequence of exendin-4 for binding to the GLP-1 receptor and for biological activity. Regul Pept 114:153–158. 12832104 10.1016/s0167-0115(03)00120-4 PMC10031803

[B13] Fang Z Chen S Manchanda Y Bitsi S Pickford P David A Shchepinova MM Corrêa IR Jr Hodson DJ Broichhagen J, (2020a) Ligand-specific factors influencing GLP-1 receptor post-endocytic trafficking and degradation in pancreatic beta cells. Int J Mol Sci 21:8404. 10.3390/ijms21218404 PMC7664906 33182425

[B14] Fang Z Chen S Pickford P Broichhagen J Hodson DJ Corrêa IR Jr Kumar S Görlitz F Dunsby C French PMW, (2020b) The influence of peptide context on signaling and trafficking of glucagon-like peptide-1 receptor biased agonists. ACS Pharmacol Transl Sci 3:345–360. 32296773 10.1021/acsptsci.0c00022 PMC7155199

[B15] Fletcher MM Halls ML Zhao P Clydesdale L Christopoulos A Sexton PM Wootten D (2018) Glucagon-like peptide-1 receptor internalisation controls spatiotemporal signalling mediated by biased agonists. Biochem Pharmacol 156:406–419. 30195733 10.1016/j.bcp.2018.09.003

[B16] Fox CB Wayment JR Myers GA Endicott SK Harris JM (2009) Single-molecule fluorescence imaging of peptide binding to supported lipid bilayers. Anal Chem 81:5130–5138. 19480398 10.1021/ac9007682

[B17] Fremaux J Venin C Mauran L Zimmer R Koensgen F Rognan D Bitsi S Lucey MA Jones B Tomas A, (2019) Ureidopeptide GLP-1 analogues with prolonged activity *in vivo via* signal bias and altered receptor trafficking. Chem Sci (Camb) 10:9872–9879. 10.1039/c9sc02079a PMC6977461 32015811

[B18] Girada SB Kuna RS Bele S Zhu Z Chakravarthi NR DiMarchi RD Mitra P (2017) Gαs regulates glucagon-like peptide 1 receptor-mediated cyclic AMP generation at Rab5 endosomal compartment. Mol Metab 6:1173–1185. 29031718 10.1016/j.molmet.2017.08.002 PMC5641683

[B19] Graaf Cd Donnelly D Wootten D Lau J Sexton PM Miller LJ Ahn J-M Liao J Fletcher MM Yang D, (2016) Glucagon-like peptide-1 and its class B g protein-coupled receptors: a long march to therapeutic successes. Pharmacol Rev 68:954–1013. 27630114 10.1124/pr.115.011395 PMC5050443

[B20] Guariguata L Whiting DR Hambleton I Beagley J Linnenkamp U Shaw JE (2014) Global estimates of diabetes prevalence for 2013 and projections for 2035. Diabetes Res Clin Pract 103:137–149. 24630390 10.1016/j.diabres.2013.11.002

[B21] Harikumar KG Wootten D Pinon DI Koole C Ball AM Furness SGB Graham B Dong M Christopoulos A Miller LJ, (2012) Glucagon-like peptide-1 receptor dimerization differentially regulates agonist signaling but does not affect small molecule allostery. Proc Natl Acad Sci USA 109:18607–18612. 23091034 10.1073/pnas.1205227109 PMC3494884

[B22] Hohmeier HE Mulder H Chen G Henkel-Rieger R Prentki M Newgard CB (2000) Isolation of INS-1-derived cell lines with robust ATP-sensitive K+ channel-dependent and -independent glucose-stimulated insulin secretion. Diabetes 49:424–430. 10868964 10.2337/diabetes.49.3.424

[B23] Huang B Perroud TD Zare RN (2004) Photon counting histogram: one-photon excitation. ChemPhysChem 5:1523–1531. 15535551 10.1002/cphc.200400176

[B24] Inoue A Raimondi F Kadji FMN Singh G Kishi T Uwamizu A Ono Y Shinjo Y Ishida S Arang N, (2019) Illuminating G-protein-coupling selectivity of GPCRs. Cell 177:1933–1947.e25. 31160049 10.1016/j.cell.2019.04.044 PMC6773469

[B25] Irannejad R Tomshine JC Tomshine JR Chevalier M Mahoney JP Steyaert J Rasmussen SGF Sunahara RK El-Samad H Huang B, (2013) Conformational biosensors reveal GPCR signalling from endosomes. Nature 495:534–538. 23515162 10.1038/nature12000 PMC3835555

[B26] Jaccard N Griffin LD Keser A Macown RJ Super A Veraitch FS Szita N (2014) Automated method for the rapid and precise estimation of adherent cell culture characteristics from phase contrast microscopy images. Biotechnol Bioeng 111:504–517. 24037521 10.1002/bit.25115 PMC4260842

[B27] Jones B Buenaventura T Kanda N Chabosseau P Owen BM Scott R Goldin R Angkathunyakul N Corrêa IR Jr Bosco D, (2018) Targeting GLP-1 receptor trafficking to improve agonist efficacy. Nat Commun 9:1602. 29686402 10.1038/s41467-018-03941-2 PMC5913239

[B28] Kenakin T Watson C Muniz-Medina V Christopoulos A Novick S (2012) A simple method for quantifying functional selectivity and agonist bias. ACS Chem Neurosci 3:193–203. 22860188 10.1021/cn200111m PMC3369801

[B29] Knudsen LB Lau J (2019) The discovery and development of liraglutide and semaglutide. Front Endocrinol (Lausanne) 10:155. 31031702 10.3389/fendo.2019.00155 PMC6474072

[B30] Kolterman OG Buse JB Fineman MS Gaines E Heintz S Bicsak TA Taylor K Kim D Aisporna M Wang Y, (2003) Synthetic exendin-4 (exenatide) significantly reduces postprandial and fasting plasma glucose in subjects with type 2 diabetes. J Clin Endocrinol Metab 88:3082–3089. 12843147 10.1210/jc.2002-021545

[B31] Koole C Reynolds CA Mobarec JC Hick C Sexton PM Sakmar TP (2017) Genetically encoded photocross-linkers determine the biological binding site of exendin-4 peptide in the N-terminal domain of the intact human glucagon-like peptide-1 receptor (GLP-1R). J Biol Chem 292:7131–7144. 28283573 10.1074/jbc.M117.779496 PMC5409479

[B32] Kucherak OA Oncul S Darwich Z Yushchenko DA Arntz Y Didier P Mély Y Klymchenko AS (2010) Switchable nile red-based probe for cholesterol and lipid order at the outer leaflet of biomembranes. J Am Chem Soc 132:4907–4916. 20225874 10.1021/ja100351w

[B33] Kuna RS Girada SB Asalla S Vallentyne J Maddika S Patterson JT Smiley DL DiMarchi RD Mitra P (2013) Glucagon-like peptide-1 receptor-mediated endosomal cAMP generation promotes glucose-stimulated insulin secretion in pancreatic β-cells. Am J Physiol Endocrinol Metab 305:E161–E170. 23592482 10.1152/ajpendo.00551.2012

[B34] Lau J Bloch P Schäffer L Pettersson I Spetzler J Kofoed J Madsen K Knudsen LB McGuire J Steensgaard DB, (2015) Discovery of the once-weekly glucagon-like peptide-1 (GLP-1) analogue semaglutide. J Med Chem 58:7370–7380. 26308095 10.1021/acs.jmedchem.5b00726

[B35] Lee JG Ryu JH Kim S-M Park M-Y Kim S-H Shin YG Sohn J-W Kim HH Park Z-Y Seong JY, (2018) Replacement of the C-terminal Trp-cage of exendin-4 with a fatty acid improves therapeutic utility. Biochem Pharmacol 151:59–68. 29522713 10.1016/j.bcp.2018.03.004

[B36] Levoye A Zwier JM Jaracz-Ros A Klipfel L Cottet M Maurel D Bdioui S Balabanian K Prézeau L Trinquet E, (2015) A broad G protein-coupled receptor internalization assay that combines SNAP-Tag labeling, diffusion-enhanced resonance energy transfer, and a highly emissive terbium cryptate. Front Endocrinol (Lausanne) 6:167. 26617570 10.3389/fendo.2015.00167 PMC4638144

[B37] Lucey M Pickford P Bitsi S Minnion J Ungewiss J Schoeneberg K Rutter GA Bloom SR Tomas A Jones B (2020) Disconnect between signalling potency and in vivo efficacy of pharmacokinetically optimised biased glucagon-like peptide-1 receptor agonists. Mol Metab 37:100991. 32278079 10.1016/j.molmet.2020.100991 PMC7262448

[B38] Mahammad S Parmryd I (2015) Cholesterol depletion using methyl-β-cyclodextrin. Methods Mol Biol 1232:91–102. 25331130 10.1007/978-1-4939-1752-5_8

[B39] Malm-Erjefält M Bjørnsdottir I Vanggaard J Helleberg H Larsen U Oosterhuis B van Lier JJ Zdravkovic M Olsen AK (2010) Metabolism and excretion of the once-daily human glucagon-like peptide-1 analog liraglutide in healthy male subjects and its in vitro degradation by dipeptidyl peptidase IV and neutral endopeptidase. Drug Metab Dispos 38:1944–1953. 20709939 10.1124/dmd.110.034066

[B40] Manchanda Y Bitsi S Kang Y Jones B Tomas A (2021) Spatiotemporal control of GLP-1 receptor activity. Curr Opin Endocr Metab Res 16:19–27.

[B41] Martineau M Somasundaram A Grimm JB Gruber TD Choquet D Taraska JW Lavis LD Perrais D (2017) Semisynthetic fluorescent pH sensors for imaging exocytosis and endocytosis. Nat Commun 8:1412. 29123102 10.1038/s41467-017-01752-5 PMC5680258

[B42] Masuho I Ostrovskaya O Kramer GM Jones CD Xie K Martemyanov KA (2015) Distinct profiles of functional discrimination among G proteins determine the actions of G protein-coupled receptors. Sci Signal 8:ra123. 26628681 10.1126/scisignal.aab4068 PMC4886239

[B43] Mystek P Dutka P Tworzydło M Dziedzicka-Wasylewska M Polit A (2016) The role of cholesterol and sphingolipids in the dopamine D_1_ receptor and G protein distribution in the plasma membrane. Biochim Biophys Acta 1861:1775–1786. 27570114 10.1016/j.bbalip.2016.08.015

[B44] Neidigh JW Fesinmeyer RM Prickett KS Andersen NH (2001) Exendin-4 and glucagon-like-peptide-1: NMR structural comparisons in the solution and micelle-associated states. Biochemistry 40:13188–13200. 11683627 10.1021/bi010902s

[B45] Novikoff A O’Brien SL Bernecker M Grandl G Kleinert M Knerr PJ Stemmer K Klingenspor M Zeigerer A DiMarchi R, (2021) Spatiotemporal GLP-1 and GIP receptor signaling and trafficking/recycling dynamics induced by selected receptor mono- and dual-agonists. Mol Metab 49:101181. 33556643 10.1016/j.molmet.2021.101181 PMC7921015

[B46] Nuber S Zabel U Lorenz K Nuber A Milligan G Tobin AB Lohse MJ Hoffmann C (2016) β-Arrestin biosensors reveal a rapid, receptor-dependent activation/deactivation cycle. Nature 531:661–664. 27007855 10.1038/nature17198 PMC5157050

[B47] Oishi A Dam J Jockers R (2019) β-Arrestin-2 BRET biosensors detect different β-arrestin-2 conformations in interaction with GPCRs. ACS Sens 5:57–64. 31849219 10.1021/acssensors.9b01414

[B48] Olsen RHJ DiBerto JF English JG Glaudin AM Krumm BE Slocum ST Che T Gavin AC McCorvy JD Roth BL, (2020) TRUPATH, an open-source biosensor platform for interrogating the GPCR transducerome. Nat Chem Biol 16:841–849. 32367019 10.1038/s41589-020-0535-8 PMC7648517

[B49] Onaran HO Ambrosio C Uğur Ö Madaras Koncz E Grò MC Vezzi V Rajagopal S Costa T (2017) Systematic errors in detecting biased agonism: Analysis of current methods and development of a new model-free approach. Sci Rep 7:44247. 28290478 10.1038/srep44247 PMC5349545

[B50] Peng T Thorn K Schroeder T Wang L Theis FJ Marr C Navab N (2017) A BaSiC tool for background and shading correction of optical microscopy images. Nat Commun 8:14836–14837. 28594001 10.1038/ncomms14836 PMC5472168

[B51] Pickford P Lucey M Fang Z Bitsi S de la Serna JB Broichhagen J Hodson DJ Minnion J Rutter GA Bloom SR, (2020) Signalling, trafficking and glucoregulatory properties of glucagon‐like peptide‐1 receptor agonists exendin‐4 and lixisenatide. Br J Pharmacol 177:3905–3923. 32436216 10.1111/bph.15134 PMC7429481

[B52] Roed SN Nøhr AC Wismann P Iversen H Bräuner-Osborne H Knudsen SM Waldhoer M (2015) Functional consequences of glucagon-like peptide-1 receptor cross-talk and trafficking. J Biol Chem 290:1233–1243. 25451942 10.1074/jbc.M114.592436 PMC4294488

[B53] Roed SN Wismann P Underwood CR Kulahin N Iversen H Cappelen KA Schäffer L Lehtonen J Hecksher-Soerensen J Secher A, (2014) Real-time trafficking and signaling of the glucagon-like peptide-1 receptor. Mol Cell Endocrinol 382:938–949. 24275181 10.1016/j.mce.2013.11.010

[B54] Sage D Donati L Soulez F Fortun D Schmit G Seitz A Guiet R Vonesch C Unser M (2017) DeconvolutionLab2: an open-source software for deconvolution microscopy. Methods 115:28–41. 28057586 10.1016/j.ymeth.2016.12.015

[B55] Surdo NC Berrera M Koschinski A Brescia M Machado MR Carr C Wright P Gorelik J Morotti S Grandi E, (2017) FRET biosensor uncovers cAMP nano-domains at β-adrenergic targets that dictate precise tuning of cardiac contractility. Nat Commun 8:15031. 28425435 10.1038/ncomms15031 PMC5411486

[B56] Thornton K Gorenstein DG (1994) Structure of glucagon-like peptide (7-36) amide in a dodecylphosphocholine micelle as determined by 2D NMR. Biochemistry 33:3532–3539. 8142350 10.1021/bi00178a009

[B57] Tiulpakov A White CW Abhayawardana RS See HB Chan AS Seeber RM Heng JI Dedov I Pavlos NJ Pfleger KDG (2016) Mutations of vasopressin receptor 2 including novel L312S have differential effects on trafficking. Mol Endocrinol 30:889–904. 27355191 10.1210/me.2016-1002 PMC4965841

[B58] Tomas A Jones B Leech C (2019) New insights into beta-cell GLP-1 receptor and cAMP signaling. J Mol Biol 432:1347–1366. 31446075 10.1016/j.jmb.2019.08.009

[B59] Trier S Linderoth L Bjerregaard S Andresen TL Rahbek UL (2014) Acylation of Glucagon-like peptide-2: interaction with lipid membranes and in vitro intestinal permeability. PLoS One 9:e109939. 25295731 10.1371/journal.pone.0109939 PMC4190408

[B60] van der Westhuizen ET Breton B Christopoulos A Bouvier M (2014) Quantification of ligand bias for clinically relevant β2-adrenergic receptor ligands: implications for drug taxonomy. Mol Pharmacol 85:492–509. 24366668 10.1124/mol.113.088880

[B61] Vilardaga J-P Jean-Alphonse FG Gardella TJ (2014) Endosomal generation of cAMP in GPCR signaling. Nat Chem Biol 10:700–706. 25271346 10.1038/nchembio.1611 PMC4417940

[B62] Villar VAM Cuevas S Zheng X Jose PA (2016) Localization and signaling of GPCRs in lipid rafts. Methods Cell Biol 132:3–23. 26928536 10.1016/bs.mcb.2015.11.008

[B63] Wan Q Okashah N Inoue A Nehmé R Carpenter B Tate CG Lambert NA (2018) Mini G protein probes for active G protein-coupled receptors (GPCRs) in live cells. J Biol Chem 293:7466–7473. 29523687 10.1074/jbc.RA118.001975 PMC5949987

[B64] Widmann C Dolci W Thorens B (1995) Agonist-induced internalization and recycling of the glucagon-like peptide-1 receptor in transfected fibroblasts and in insulinomas. Biochem J 310:203–214. 7646446 10.1042/bj3100203 PMC1135874

[B65] Willard FS Douros JD Gabe MB Showalter AD Wainscott DB Suter TM Capozzi ME van der Velden WJ Stutsman C Cardona GR, (2020) Tirzepatide is an imbalanced and biased dual GIP and GLP-1 receptor agonist. JCI Insight 5:1202. 10.1172/jci.insight.140532 PMC7526454 32730231

[B66] Wu F Yang L Hang K Laursen M Wu L Han GW Ren Q Roed NK Lin G Hanson MA, (2020) Full-length human GLP-1 receptor structure without orthosteric ligands. Nat Commun 11:1272. 32152292 10.1038/s41467-020-14934-5 PMC7062719

[B67] Zhang H Sturchler E Zhu J Nieto A Cistrone PA Xie J He L Yea K Jones T Turn R, (2015) Autocrine selection of a GLP-1R G-protein biased agonist with potent antidiabetic effects. Nat Commun 6:8918. 26621478 10.1038/ncomms9918 PMC4686834

[B68] Zhang J Bai Q Pérez-Sánchez H Shang S An X Yao X (2019) Investigation of ECD conformational transition mechanism of GLP-1R by molecular dynamics simulations and Markov state model. Phys Chem Chem Phys 21:8470–8481. 30957116 10.1039/c9cp00080a

[B69] Zhao P Liang Y-L Belousoff MJ Deganutti G Fletcher MM Willard FS Bell MG Christe ME Sloop KW Inoue A, (2020) Activation of the GLP-1 receptor by a non-peptidic agonist. Nature 577:432–436. 31915381 10.1038/s41586-019-1902-z

[B70] Ziomkiewicz I Loman A Klement R Fritsch C Klymchenko AS Bunt G Jovin TM Arndt-Jovin DJ (2013) Dynamic conformational transitions of the EGF receptor in living mammalian cells determined by FRET and fluorescence lifetime imaging microscopy. Cytometry A 83:794–805. 23839800 10.1002/cyto.a.22311

